# Oral proton‐reprogrammed nanomedicine to break the inflammatory bowel disease‐*Clostridium difficile* infection vicious cycle

**DOI:** 10.1002/imt2.70112

**Published:** 2026-02-20

**Authors:** Wensheng Chen, Yuntao Zhang, Jue Wang, Yiming Li, Hao Wu, Qiong Huang, Wei Wu, Kelong Ai

**Affiliations:** ^1^ Department of Pharmacy Xiangya Hospital, Central South University Changsha China; ^2^ National Clinical Research Center for Geriatric Diseases, Xiangya Hospital Central South University Changsha China; ^3^ Xiangya School of Pharmaceutical Sciences, Central South University Changsha China; ^4^ Hunan Provincial Key Laboratory of Cardiovascular Research, Xiangya School of Pharmaceutical Sciences, Central South University Changsha China; ^5^ Department of Geriatric Surgery Xiangya Hospital, Central South University Changsha China; ^6^ Department of Gastroenterology Xiangya Hospital, Central South University Changsha China; ^7^ Department of General Surgery Xiangya Hospital, Central South University Changsha China; ^8^ Key Laboratory of Biological Nanotechnology of National Health Commission Changsha China; ^9^ Key Laboratory of Aging‐related Bone and Joint Diseases Prevention and Treatment, Ministry of Education, Xiangya Hospital, Central South University Changsha China

**Keywords:** *Clostridium difficile* infection, inflammatory bowel disease, metabolic reprogramming, nanomedicine, uncoupling protein 2

## Abstract

*Clostridium difficile* infection (CDI) is a major complication of inflammatory bowel disease (IBD), but the complicated treatment strategy of antimicrobial drugs and immunosuppressive drugs cannot effectively control the syndrome of IBD and CDI. Glycolysis in intestinal epithelial cells (IECs) leads to excessive production of lactic acid, which is considered to be a key factor in the destruction of the intestinal barrier by toxin B of *Clostridium difficile* (TcdB). In this study, we revealed that uncoupling protein 2 (UCP2) was highly expressed in IECs, and disrupted mitochondrial function to reprogram metabolism toward glycolysis, thereby weakening the resistance of IECs to TcdB. We developed an oral proton‐reprogrammed nanomedicine (OPR) to convert the metabolism of IECs from glycolysis to oxidative phosphorylation, which can effectively alleviate IBD and CDI syndromes. OPR blocks the rapid delivery of protons to the mitochondrial matrix induced by UCP2, which restores mitochondrial membrane potential (MMP) and drives protons to reenter the matrix through ATP synthase, mediating the recovery of mitochondrial function. In mouse IBD and IBD complicated CDI (IBD‐CDI) models, OPR selectively targeted inflammatory colonic lesions, alleviated mitochondria‐dependent pyroptosis of IECs, inhibited macrophage STING pathway activation, and reduced lactic acid levels. Especially in the treatment of IBD‐CDI syndrome, the therapeutic effect of OPR is far better than the combination of antibiotics and immunosuppressive drugs. In summary, our study establishes UCP2‐mediated mitochondrial disability as a key mechanism for the functioning of TcdB and suggests that IEC metabolic reprogramming could be a therapeutic target.

## Highlights

- This study reveals that UCP2 is highly expressed in intestinal epithelial cells (IECs) of inflammatory bowel disease (IBD) and induces mitochondria dysfunction. The UCP2‐induced mitochondria damage promotes IEC acidification and aggravates the toxicity of TcdB.
- This study develops a new oral nanomedicine, OPR, which could sequentially target intestinal lesion, mitochondria, and UCP2 to treat IBD‐CDI.
- This study may provide a new strategy to alleviate IBD‐CDI.

## Graphical abstract

Research on the mechanism of OPR restoring intestinal epithelial cell (IEC) structure and function in inflammatory bowel disease (IBD) treatment. (A) Schematic diagram of OPR structure and the rate of GP release under different conditions. (B) Schematic diagram of OPR targeting IBD colonic lesions through charge interaction. (C) After entering cells, OPR targets outer mitochondrial membrane (OMM) proteins and releases GP. The released GP restores MMP by inhibiting UCP2 protein, thereby restoring mitochondrial function. (D) ① ROS is activated during mitochondria damage through GSDME signal and promotes pyroptosis in IECs. ② Mitochondrial damage and release of mtDNA from IECs are sensed by macrophages, and activate the STING pathway. ③ *Clostridium difficile* disrupts the cytoskeletal network of IECs through the release of TcdB, which mediates the glycosylation of Rac1. ④ OPR targets (OMM) proteins to clear ROS while releasing UCP2 inhibitor (GP) to restore mitochondrial function and inhibit cytoplasmic acidification to resist the toxic effects of TcdB.

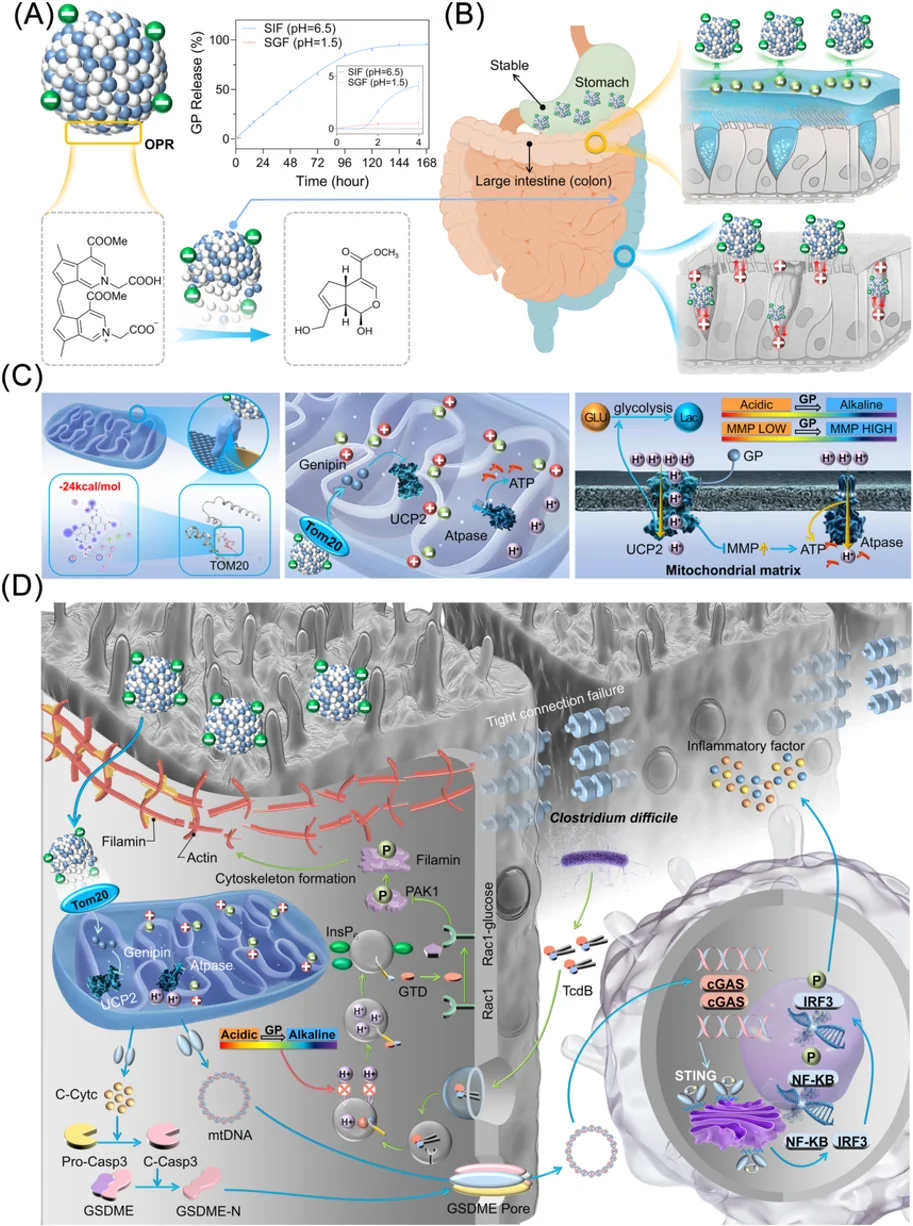

## INTRODUCTION

The prevalence of inflammatory bowel disease (IBD) continues to rise worldwide, currently affecting about 7 million patients, and has become a major challenge in the field of global public health [1]. The IBD treatment regimen not only has a low remission rate, but more importantly, IBD is prone to co‐infection with multiple pathogens, and *Clostridium difficile* infection (CDI) accounts for 97% of them [2, 3]. IBD patients with CDI have a worse prognosis [4]. Toxin B of *Clostridium difficile* (TcdB) destroys the tight junctions between intestinal epithelial cells (IECs), thereby damaging the intestinal barrier, which facilitates a vicious cycle of “IBD‐CDI” and drives IBD to worsen and persist [5, 6]. The clinical treatment for IBD‐CDI is mainly the combination of antibiotics and first‐line IBD treatment drugs (which often include immunosuppressive agents), which inevitably lead to conflicts in the treatment of IBD symptoms and CDI symptoms and the superposition of toxic effects [4]. Antibiotics may lead to further disturbance of intestinal flora to aggravate IBD, while the immunosuppressive drugs aggravate CDI infection [7]. This bidirectional pathological interference mechanism puts patients at risk of alternating worsening of symptoms, highlighting the critical need for therapeutic strategies capable of breaking this “IBD‐CDI” cycle without exacerbating the underlying disease or infection.

Significant upregulation of uncoupling protein 2 (UCP2) expression is observed in human IBD intestinal tissue and IBD mouse models, and is positively correlated with disease severity [8]. UCP2 uncouples oxidative phosphorylation by facilitating proton influx into the mitochondrial matrix, reducing mitochondrial membrane potential (MMP) and thus reduce reactive oxygen species (ROS) levels caused by electron leakage in the mitochondrial respiratory chain. Abnormally high expression of UCP2 can cause significant dissipation of the MMP, directly reducing ATP synthesis [9]. Importantly, UCP2 causes cellular metabolic reprogramming to drive mitochondrial oxidative phosphorylation toward glycolysis, and increases the production of l‐lactic acid (L‐LA) [10]. The lack of ATP in IECs leads to failure of the monocarboxylate transporter ion pump, resulting in cytoplasmic acidification caused by L‐LA accumulation in the colon of IBD patients [11, 12, 13]. The acidified IECs have a weakened resistance to TcdB, further aggravating IBD symptoms through toxin‐mediated cytoskeletal collapse [14]. In this study, we propose for the first time that reprogramming mitochondrial proton flux by inhibiting UCP2 is a highly effective strategy for treating IBD and even alleviating IBD and CDI syndromes. Inhibition of UCP2 can inhibit pyroptosis of IECs by restoring mitochondrial function, thereby reprogram metabolism of IECs to prevent CDI‐mediated toxicity by inhibiting cytoplasmic acidification. However, simple inhibition of UCP2 can lead to increased ROS [9], whereas excessive ROS aggravates IBD [15]. Meanwhile, UCP2 is widely distributed in the human body, and non‐targeted inhibition may lead to side effects [16]. To this end, we developed an oral proton‐reprogrammed nanomedicine (OPR) for the treatment of IBD and CDI‐IBD. Unlike traditional broad‐spectrum antibiotics, nanomaterials with excellent bioavailability exhibit fewer side effects in anti‐infection and improve therapeutic efficacy through comprehensive regulation of the immune system [17]. Therefore, nanomaterials are redefining more attractive and promising IBD treatment strategies. OPR was constructed with an IBD lesion‐IEC mitochondria sequential targeted drug release system: glycine and Genipin (GP, a potent UCP2 inhibitor) were polymerized into 5–10 nm nanoparticles. Genipin has been widely studied and recognized as a specific UCP2 inhibitor, and we found that UCP2 may play an important role in IBD pathology, so we selected Genipin as the main effector component of OPR for specific inhibition of UCP2 [18]. However, Genipin itself does not have the ability to target the intestine, so it needs to be structurally modified, and we chose glycine as another component for the following reasons: ① The nanoparticles synthesized by Genipin and glycine have a negative charge on the surface and can target IBD intestinal lesions through charge attraction. ② Glycine is a natural amino acid with the smallest molecular weight and simplest structure. Its unique physicochemical properties, such as high solubility, zwitterionic properties, buffering capacity, chemical stability, and low toxicity, make it an indispensable and versatile excipient in the biopharmaceutical field. It is widely used in protein drug preparations, intravenous injections, diagnostic reagents, and cell cultures, mainly playing key roles such as stabilizers, buffers, isotonic regulators, and protective agents [19, 20]. The intestine usually only allows molecules with a molecular weight of less than 3 kDa to pass through, but cannot pass through high molecular weight nanoparticles. Accordingly, OPR can significantly reduce the side effects of systemic distribution.

After oral administration, OPR can maintain stability in the highly acidic environment of the stomach, while slowly releasing the active ingredient, GP, in the slightly acidic environment of IBD lesions for up to 6 days. Negatively charged OPRs specifically target positively charged inflamed areas of the IBD colon and away from negatively charged healthy intestinal segments. More importantly, OPR preferentially associates with outer mitochondrial membrane proteins, including TOM20/TOM34/VDACs, and releases GP. Specifically, GP can inhibit UCP2‐mediated proton backflow and restore MMP, which drives protons to reenter the mitochondrial matrix through ATP synthase and mediates ATP synthesis. Accordingly, OPR can restore mitochondrial function in IECs, reduce mitochondria‐dependent pyroptosis and mitochondria DNA (mtDNA) release. Consequently, OPR can drive the metabolism of IECs to shift from glycolysis to oxidative phosphorylation, significantly reducing the production of L‐LA, which inhibits cell acidification and makes IECs resistant to the TcdB. Critically, while UCP2 inhibition typically induces ROS upregulation, OPR effectively eliminates multiple ROS species, preventing this detrimental effect. In a mouse IBD model, OPR can alleviate pyroptosis of IECs, subsequently inhibit activation of the macrophage cGAS‐STING pathway, and demonstrate a therapeutic effect far superior to that of first‐line clinical therapeutic drugs, including 5‐Aminosalicylic Acid (5‐ASA), Dexamethasone (DEX), JAK1 inhibitor Upadacitinib (UPA), and TNF‐α monoclonal antibody Infliximab (INF). Moreover, OPR reduces L‐LA levels and inhibits cell acidification through metabolic reprogramming to increase the tolerance of IECs to TcdB and effectively alleviating IBD‐CDI complications. In summary, this study provides a new paradigm for the treatment of IBD‐CDI syndrome from the perspective of inhibiting IEC pyroptosis and increasing toxin tolerance of IECs.

## RESULTS

### UCP2 in IBD and synthesis of OPR targeting IBD lesion

To confirm the role of UCP2 in IBD, we performed a comprehensive analysis of the human source dataset [GEO]GSE179285 from the GEO database. There were significant differences in gene expression profiles at the IBD group (inflammatory colon) compared to the Control group (non‐inflammatory colon). The volcano plot showed a total of 712 differentially expressed genes (DEGs) in the IBD group versus the Control group, of which 494 DEGs were upregulated, and 218 DEGs were downregulated (Figure S1A). We performed genomic database Kyoto Encyclopedia of Genes and Genomes (KEGG) enrichment analysis on the 712 DEGs and found that they were mainly enriched in the pathways related to ROS, cytoskeletal proteins, inflammatory response, intestinal tight junctions, and intestinal flora (Figure 1A). We then performed gene set enrichment analysis on all genes and found that the glycolysis pathway (Figure S1B) and ROS‐related pathway were significantly upregulated in IBD patients (Figure S1C), whereas the cytoskeletal protein regulation of the actin cytoskeleton pathway was significantly downregulated in IBD patients (Figure S1D). Importantly, the dataset showed that *UCP2*, which is glycolysis and ROS‐related, was significantly upregulated in the colon of IBD patients compared to healthy controls (Figure S2A). Subsequently, we collected colon tissue samples from volunteers to further investigate IBD pathology. As shown in Figure S2B, the level of L‐LA in the colon from IBD patients was significantly increased, more than 2.5 times that of non‐inflamed colonic tissue from colorectal cancer (CRC) patients (used as healthy controls). H&E staining showed that the integrity of the intestinal barrier was disrupted in the colon tissues of IBD patients (Figure S2C). Transmission electron microscopy (TEM) results showed that IEC tight junctions were collapsed and mitochondrial morphology was swollen with loss of lining in IBD patients (Figure S2D). Consistent with the dataset, the co‐staining of UCP2 and Epithelial cell adhesion molecule (EpCAM, an IEC marker) showed a significant increase of UCP2 in IEC in the colon from IBD patients (Figure 1B). We stained the colon tissue for ROS by dihydroethidium (DHE), and observed a significant elevation of ROS in the colon of IBD patients (Figure 1C), which was consistent with the results of the bioinformatics analysis. Further fluorescence staining showed that mtDNA (dsDNA‐labeled) was released from mitochondria (TOM20‐labeled) in large quantities in the IECs of patients with IBD (Figure 1D), and skeletal proteins of IECs of IBD patients were significantly collapsed (Figure 1E), and Cleaved‐Caspase3 (C‐Casp3) and Gasdermin E (GSDME) were highly co‐expressed in the IECs of IBD patients, suggesting the occurrence of cellular pyroptosis (Figure 1F).

**Figure 1:**
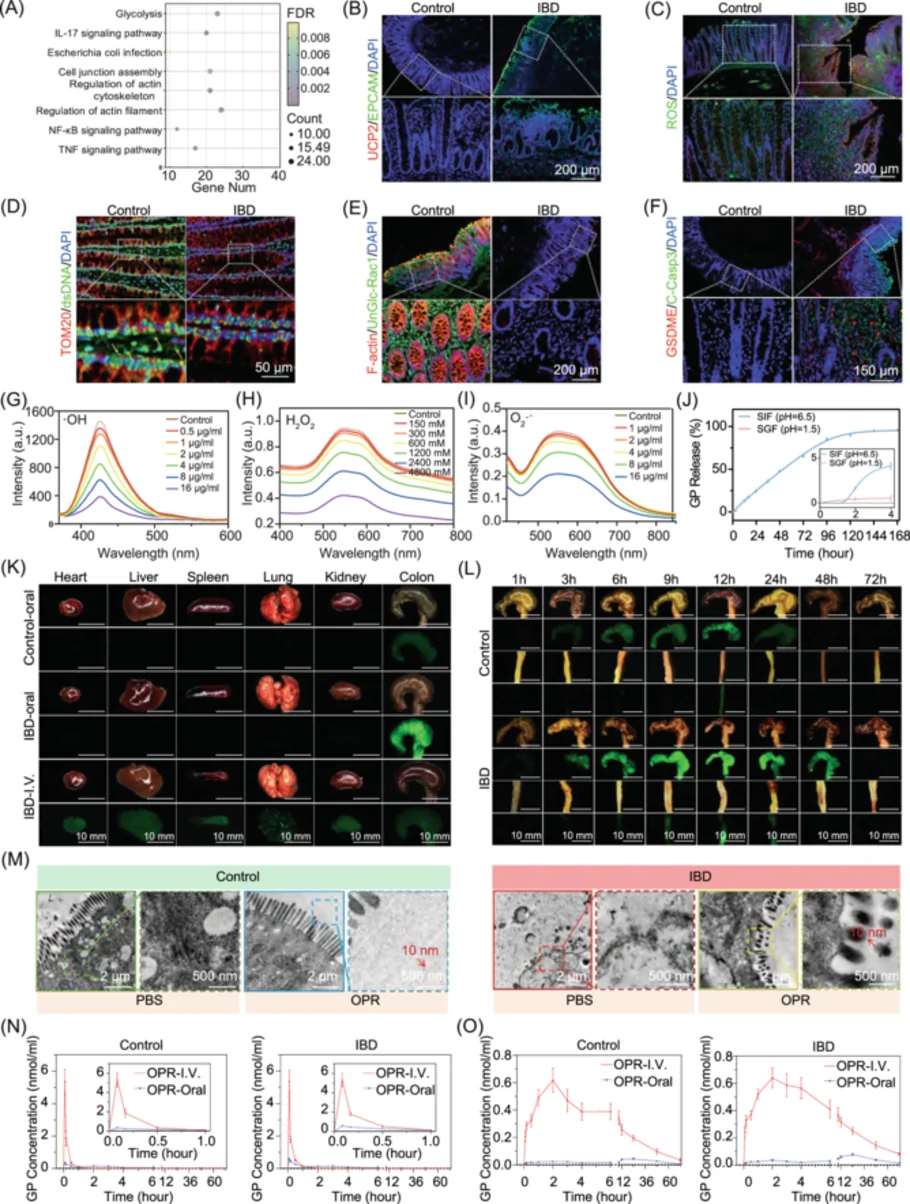
Synthesis and characterization of OPR targeting excessive ROS in IBD. (A) KEGG enrichment analysis of DEGs between Control group and IBD group (FDR < 0.05). (Control *n* = 20, IBD *n* = 16). (B–F) Representative image of immunofluorescent staining of UCP2‐EpCAM (B), ROS (C), TOM20‐dsDNA (D), F‐actin/UnGlc‐Rac1 (E), and GSDME‐C‐Casp3 (F) in colon tissues of Control group and IBD group. Scale bar is 200, 200, 50, 200, 150 µm, respectively. (Control *n* = 3, IBD *n* = 3). (G) ·OH scavenging ability of different doses of OPR. (H) H_2_O_2_ scavenging ability of different doses of OPR. (I) O_2_·^−^ scavenging ability of different doses of OPR. (J) Folding line graph of the amount of GP released by OPR in SGF or SIF environment in 4 or 144 h (*n* = 3). (K) Representative image of fluorescence in vivo of main organs (heart, liver, spleen, lung, kidney, and colon) in IBD and healthy mice after oral and intravenous administration of OPR‐FITC. Scale bar is 10 mm (*n* = 3). (L) Representative image of intestinal fluorescence of IBD mice within 72 h after oral administration of OPR‐FITC. Scale bar is 10 mm (*n* = 3). (M) TEM observation of the targeted retention of OPR on the intestinal epithelium of healthy mice and IBD mice (green frame: healthy mice; blue frame: healthy mice orally administered OPR; red frame: IBD mice; yellow frame: IBD mice orally administered OPR). Scale bars are 2 µm and 500 nm (*n* = 3). (N, O) Time‐dependent linear diagram of GP concentration in the peripheral blood of healthy mice or IBD mice after oral or intravenous injection of OPR or GP. Healthy and IBD mice after oral or intravenous injection of GP (N). Healthy and IBD mice after oral or intravenous injection of OPR (O) (*n* = 6). C‐Casp3, cleaved‐caspase3; DEG, differentially expressed genes; dsDNA, double‐stranded DNA; EpCAM, epithelial cell adhesion molecule; IBD, inflammatory bowel disease; FITC, fluorescein isothiocyanate; GSDME, gasdermin E; GP, genipin; KEGG, Kyoto Encyclopedia of Genes and Genomes database; OPR, oral proton‐reprogrammed nanomedicine; ROS, reactive oxygen species; SGF, simulated gastric fluid; SIF, simulated intestinal fluid; TOM20, translocase of outer mitochondrial membrane 20; TEM, transmission electron microscopy; UCP2, uncoupling protein 2; UnGlc‐Rac1, unglycosylated‐Ras‐related C3 botulinum toxin substrate 1.

GP is a biological cross‐linking agent, resulting in cross‐linking with digestive tract proteins after oral administration and failing to reach the intestines [21]. To solve the above difficulties, we opened a ring of GP to form a hemiacetal intermediate and then polymerized it with glycine to form OPR in a strongly acidic aqueous solution, and the resulting OPR closed the GP hemiacetal site to prevent cross‐linking with proteins (Figure S3A). Through zeta potential measurement, OPR showed a strong negative charge under a physiological condition (pH = 7.4, PBS) and an environment of simulated gastric fluid (pH = 1.5, SGF) (Figure S3B). TEM showed that OPR has a monodisperse spherical structure with an average diameter of about 5–10 nm, and OPR did not change its morphology and size after immersion in SGF for 4 h (the incubation time of OPR in SGF was 4 h, simulating the duration of gastric emptying) (Figure S3C). X‐ray photoelectron spectroscopy (XPS) showed OPR contained both C, O, and N indicating that GP and glycine were successfully polymerized into OPR (Figure S3D). Through the XPS fine spectrum, C1s of OPR can be divided into three parts, namely C─C/C═C/C─H (the peak located at 284.8 eV), C─O/C─N (the peak located at 286.2 eV), and O─C═O (the peak located at 289.1 eV) (Figure S3E). The peak located at 401.1 eV in OPR's N1s spectrum was attributed to the graphite nitrogen produced by the reaction of the cyclic hemiacetal structure in the pentene dihydropyran and amino group, confirming the successful cross‐linking of GP with glycine via the Schiff base reaction, in addition to the inclusion of an amine peak at 399.3/402.8 eV in OPR (Figure S3F). The O1s spectrum of the OPR (Figure S3G) corresponded to hydroxyl (C–OH, 532.4 eV), carbonyl oxygen (C═O, 531.1 eV), and carboxyl oxygen (O─C═O, 533.9 eV). SGF treatment did not change the XPS spectrum of OPR (Figure S3H–J). The existence of abundant antioxidant groups, such as hydroxyl groups, provided the possibility for OPR to strongly eliminate various ROS. As expected, OPR showed excellent ·OH (Figure 1G), H_2_O_2_ (Figure 1H), O_2_·^−^ (Figure 1I) scavenging ability; the scavenging ability of SGF‐OPR for ·OH, H_2_O_2_, and O_2_·^−^ was not significantly impaired after SGF treatment (Figure S3K–M). Through the nitroblue tetrazolium method (NBT) assay, OPR was able to effectively scavenge O_2_·^−^, and its pseudo‐superoxide dismutase (SOD) activity was approximately 156.3 U/mg (Figure 1I). We tested the ability of OPR to release GP at different times under SGF or simulated intestinal fluid (SIF) (pH = 6.5, SIF) using high‐performance liquid chromatography‐mass spectrometry (LC‐MS), respectively. OPR had a higher GP release efficiency in SIF; on the contrary, the release rate of OPR was much lower in SGF (Figure 1J). In addition, the OPR solution is blue–purple while the GP solution is colorless, so we also track the OPR degradation/stability by color change (Figure S4). The color change of OPR under SIF and SGF conditions was consistent with the LC‐MS results. Based on the OPR degradation data (release GP) under SIF conditions, the calculated half‐life of OPR is about 44.95 h (Figure S5). In summary, UCP2, excessive ROS, and high L‐LA levels in IECs are closely related to the pathogenesis of IBD. These three pathological factors are involved in impairing mitochondrial function, disrupting the cytoskeleton, IEC pyroptosis, and intestinal barrier damage. We have successfully prepared OPR, which can effectively eliminate a variety of ROS and slowly release the active ingredient (GP) in the IBD lesions.

To investigate the distribution of OPR in vivo, we constructed fluorescently labeled OPR‐FITC by covalent coupling with FITC (Figure S6A). The construction of OPR‐FITC was confirmed with UV spectrum (Figure S6B). Through zeta potential measurement, OPR‐FITC did not show a significant change compared to OPR (Figure S6C). Here, the dextran sulfate sodium salt (DSS)‐induced IBD mouse model was used in our study because the pathological process and characteristics of DSS‐induced IBD are similar to those of IBD patients, especially the ulcerative colitis (UC) patients.‌ And the UC patients often exhibit high L‐LA levels and a low pH environment in the colon, conditions suitable for CDI [22]. OPR‐FITC was administered orally or intravenously to the IBD model mice with healthy mice as controls (Figure S6D). After 12 h of intravenous injection, OPR‐FITC was observed in the heart, liver, spleen, lungs, and kidneys in the major organs by stereofluorescence microscopy. As a strong contrast, through oral administration, the OPR‐FITC distribution in the colon was significantly higher than that in other tissues and organs (Figure 1K, Figure S6E). Moreover, the distribution of OPR‐FITC in the IBD colon was much higher than that in the healthy colon, and OPR‐FITC could stay in the intestines of IBD mice for up to 72 h (Figure 1L, Figure S6F) after oral administration, which provided a basis for its sustained therapeutic effect. The normal human intestinal surface has a mucus layer, which is mainly composed of mucins such as mucin 2 (MUC2) [23]. Since these mucins are usually rich in sialic acid structures and sulfate groups, which make them negatively charged [24]. A large number of inflammatory factors inhibit goblet cell differentiation and mucin synthesis at the IBD lesion site [25]. At the same time, an increase of highly positively charged proteins, such as transferrin (TF), and an accumulation of positively charged proteins released by cellular damage occur at the IBD lesion site [26, 27]. Those changes in the structure and charge of the intestinal surface make the negatively charged OPR more favorable for targeting the IBD lesion site. Further H&E staining experiments were performed on the tissue sections collected from OPR‐FITC‐enriched regions (high green fluorescence) and non‐positively stained regions (no green fluorescence) of intestinal segments. As shown in Figure S6G,H, the highly fluorescent intestinal tissue was the inflammatory intestinal lesion tissue, and the non‐fluorescent intestinal tissue was the non‐inflammatory intestinal tissue. The immunofluorescence assay showed that large amounts of negatively charged MUC2 were secreted in the colon of healthy mice, whereas MUC2 expression was low in IBD mice. In contrast, positively charged TF was highly expressed in the colon of IBD mice, whereas its expression was very weak in the colon of healthy mice (Figure S6I). To further validate the mechanism of OPR targeting against IBD lesion sites, we investigated the in vitro adhesion ability of OPR to TF protein and MUC2 protein. As shown in Figure S6J, the OPR‐FITC adhered significantly to polystyrene dishes encapsulated with TF proteins, and the fluorescence intensity was significantly higher than that of dishes encapsulated with MUC2 proteins. The interaction of OPR and GP with the above two proteins was analyzed separately using MOE software (Figure S6K). OPR had almost no interaction with the negatively charged MUC2 protein, with low binding energy (−0.5 kcal/mol). As a strong contrast, the carboxylate group of OPR was able to interact with the basic amino acids such as histidine, arginine, and lysine on the positively charged TF protein in an apparent positively and negatively charged adsorption with high binding energy (−23 kcal/mol), suggesting a strong interaction between OPR and TF. In contrast, the binding energies of GP with MUC2 (−3.6 kcal/mol) and TF (−2.8 kcal/mol) were both low, and there was no significant interaction force. To confirm that the fluorescence observed here came from OPR‐FITC instead of the abnormally accumulated FITC or GP, we synthesized GP nanoparticles similar in size to OPR and labeled GP using FITC, which were then observed/compared with OPR‐FITC in parallel. The results showed that the intestinal distribution of oral GP‐FITC in mice, especially IBD mice, was much lower than that of OPR‐FITC (Figure S7). The above results fully demonstrate that GP cannot target the intestine, while OPR has good intestinal targeting ability. To clarify the correlation between the intestinal targeting of OPR and its negative charge on its surface, we replaced the glycine in the OPR with arginine (rich in amino groups) to synthesize a nanoparticle close to OPR's size, GPA. Zeta potential detection showed a positive charge on the GPA surface (Figure S8A). The results of the protein adhesion assay showed that GPA had a low affinity for the positively charged TF protein and a high affinity for the negatively charged MUC protein, as opposed to OPR (Figure S8B). We also labeled GPA with FITC and performed stereo fluorescence observations after oral administration of mice to compare them with OPR in parallel. The results showed that the distribution of GPA‐FITC in the intestinal of IBD mice was much lower than that of OPR‐FITC, while the distribution in the intestinal of healthy mice was slightly higher than that of OPR‐FITC (Figure S8C). Further intestinal analysis showed that the distribution of GPA‐FITC in the non‐inflammatory region was significantly higher than that in the inflammatory intestinal segment, which was diametrically opposite to the distribution characteristics of OPR (Figure S8D). The above results fully demonstrate that the intestinal targeting of OPR is tightly related to its surface negative charge, and the enrichment of OPR in IBD intestinal lesions is based on the specific targeting of charge attraction, rather than passive diffusion due to barrier penetration. The above experiments demonstrate that the negative charge of OPR is an important factor in targeting IBD lesion sites. As shown in the TEM observation (Figure 1M), it is difficult for OPR to be enriched in IECs in healthy mice with normal intestinal villi intact. Whereas the sparse intestinal villi and disrupted intestinal barriers in the IBD mice make it easier for OPR to be enriched in damaged IECs.

To investigate the in vivo distribution characteristics of GP released by OPR, we used LC‐MS to detect GP levels in the peripheral blood of mice. Firstly, after direct oral administration of GP, the concentration of GP in the blood was almost undetectable, since GP is a natural cross‐linker of proteins; it cross‐links with proteins in the digestive tract before absorption into the blood. After intravenous injection of GP, the concentration of GP in the blood of mice peaked rapidly, then declined rapidly after half an hour (Figure 1N). These huge ups and downs in a short period of time also basically corresponded to the metabolism of most small‐molecule drugs in vivo. Secondly, after intravenous administration of OPR, although the slow release of GP from OPR ensured that it could maintain high blood concentrations for a longer period (24 h), it was unsuitable as an administration mode for the IBD treatment due to its lack of colonic targeting. Finally, after direct oral administration of OPR, the GP levels in blood were extremely low (<0.1 nmol/mL) (Figure 1O), indicating that OPR released GP locally in the intestine and greatly reduced the circulating metabolic loss caused by GP into the blood. Therefore, oral administration of OPR can ensure the increase of drug content specifically at the IBD lesions and reduce potential adverse effects. In conclusion, the evidence above shows that oral administration of OPR can be highly targeted to IBD lesions and can be maintained for a long time, providing a guarantee for high efficacy and low side effects of OPR.

### Therapeutic efficacy of OPR on IBD

In view of OPR's ability to efficiently target colon lesions and long‐lasting slow release of the active ingredient GP, we conducted further investigations on the efficacy of OPR. We first performed a dosage screening of OPR in IBD mice. Given that the half‐life of OPR in IBD lesions is approximately 44.95 h, we gave normal mice (*n* = 5) oral OPR 2.5, 5, 10, 20, 40, and 80 mg/kg every other day for 7 days, during which the activity status of the mice was observed, and their body weight was recorded. After 7 days, we found that mice in the 80 mg/kg dose group experienced a weight loss of about 10%, and there was no significant difference between the other dose groups and the PBS control group (Figure S9). Therefore, we preliminarily screened a safe/tolerable dose range of 2.5–40 mg/kg for oral OPR in mice. Then the IBD mice were treated with different doses of OPR (2.5 mg/kg, 5 mg/kg, 10 mg/kg) orally on Days 7, 9, and 11, respectively (Figure 2A). We used a series of indicators to verify the efficacy of OPR at different doses, including disease activity index (DAI), body weight, colon length, and intestinal epithelial injury score. The DAI is a key indicator for evaluating the status of IBD, which comprehensively evaluates weight changes, stool consistency, and fecal bleeding. The length of the mouse colon and the intestinal epidermal damage score are key indicators for evaluating the therapeutic effect of enteritis. Due to the damage of the intestinal epithelial barrier, atrophy of the mucosa, and flattening of the villus structure, the length of the mouse colon is significantly shortened under IBD conditions. The mouse intestinal injury score evaluates indicators such as edema of the intestinal mucosa, lamina propria, and muscularis, and inflammatory cell infiltration. The optimal administration dose of OPR (5 mg/kg) was determined by the body weight (Figure 2B), DAI (Figure 2C), colon length (Figure 2D, Figure S10), and H&E staining of the mouse colon and the corresponding epithelial damage score (Figure 2E,F).

**Figure 2:**
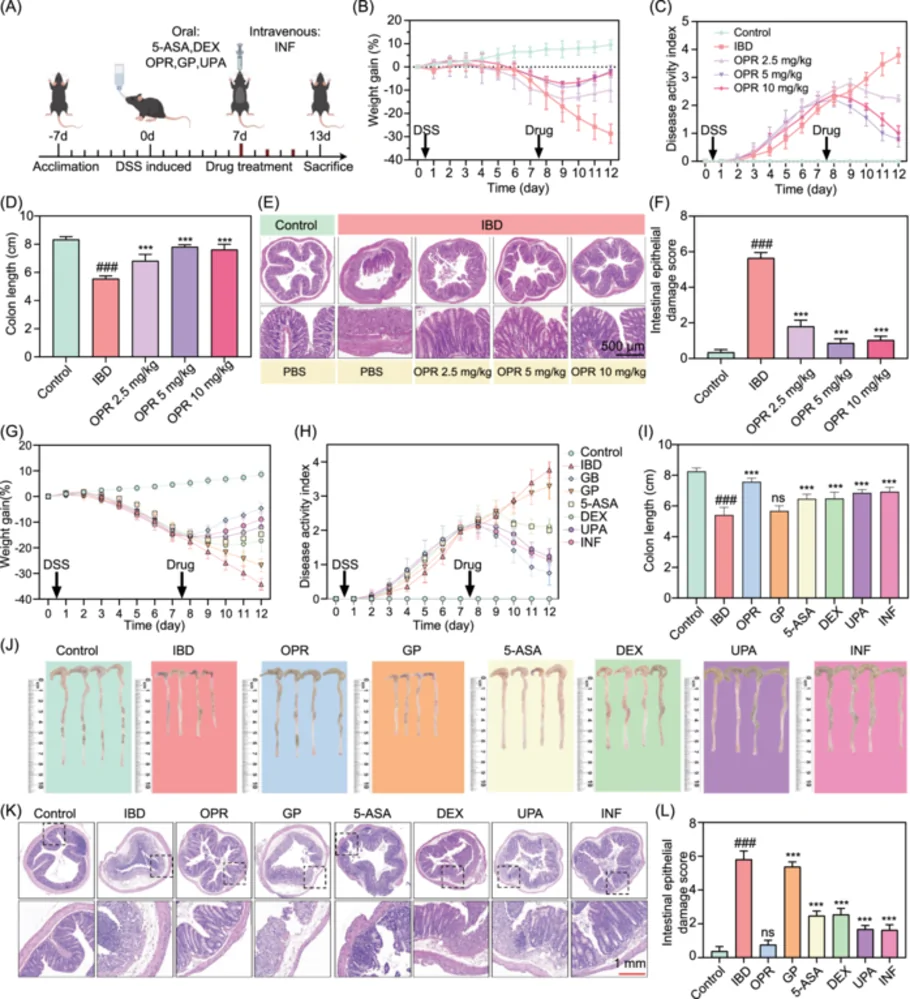
Therapeutic efficacy of oral proton‐reprogrammed (OPR) on inflammatory bowel disease (IBD). (A) Schematic diagram of IBD model establishment and different drug treatment schemes. (B–F) OPR therapeutic dose pre‐experiment. Percentage change of body weight in mice (B), DAI assessment of different treatment groups (C), (D) bar chart of colon length comparison among different groups, (E) H&E staining of intestinal sections from different groups, scale bar is 500 μm, and (F) bar chart of epithelial injury score evaluation among different groups. (*n* = 6). (G) Percentage change of body weight in mice (*n* = 8). (H) DAI assessment of different treatment groups. (*n* = 8). (I) Gross specimen of colon length comparison and (J) quantification among different groups (*n* = 8). (K) H&E staining of intestinal sections from different groups. Scale bar is 1 mm (*n* = 6). (L) Bar chart of epithelial injury score evaluation among different groups (*n* = 6). Data represent means ± S.D. Statistical significance was calculated by one‐way ANOVA with Tukey's post‐test, ^###^ *p* < 0.001 vs. Control, ***p < 0.001 vs. IBD (ns: no statistical difference compared to IBD). DAI, disease activity index; H&E, hematoxylin and eosin.

To confirm the biocompatibility and safety of OPR before the formal treatment, we performed toxicity tests in both cell and animal experiments. *In vitro*, we treated HT29 cells with 0.3–300 μg/mL (0.03–30 times the cellular therapeutic concentration) of OPR for 24/48 h, and CCK‐8 assay showed no significant change in cell viability (Figure S11A). In vivo, we tracked the dose‐effect correlation of potential toxicity response in healthy mice by gavage with a high dose (30 mg/kg, 6 times the therapeutic dose) of OPR three times within 7 days. The acute toxicity response of the drug was also tested by gavage with an ultra‐high dose (100 mg/kg, 20 times the therapeutic dose) of OPR for 24 h. Blood and tissue samples were collected for the subsequent assays (Figure S11B). Firstly, the body weight (Figure S11C), liver function indexes ALT (Figure S11D), AST (Figure S11E) and renal function indexes CRE (Figure S11F), BUN (Figure S11G) of mice in the high dose group and ultra‐high dose group showed no difference from those of the control group. The blood routine parameters WBC series (Figure S11H) (lymphocytes, monocytes, neutrophils, white blood cells), platelet series (Figure S11I) (platelets, mean platelet volume [MPV]) did not show differences among different groups. There was no significant difference in RBC series indicators (Figure S11J) (hemoglobin, mean corpuscular volume [MCV], hematokrit, mean corpuscular hemoglobin [MCH], red blood cells, mean corpuscular hemoglobin concentration [MCHC]) among all groups. Finally, H&E staining of the major organs from mice was performed to evaluate the possible toxic effects of OPR, and there were no significant morphology changes in the major organs of mice in both the high dose and ultra‐high dose groups (Figure S11K). To summarize, OPR demonstrated biocompatibility and a favorable safety both in vivo and in vitro.

After determining the OPR treatment dose, we then compared the therapeutic effects of OPR with those of 5‐ASA, DEX, UPA, and INF in the IBD mouse model. We also set up a GP group to confirm that OPR has an improved intestinal targeting ability in vivo compared with GP. After the successful construction of the IBD mouse model, we used OPR (5 mg/kg) and GP (5 mg/kg) by oral gavage every other day, 5‐ASA (30 mg/kg), DEX (1 mg/kg), and UPA (10 mg/kg) by oral gavage every day, and INF (5 mg/kg) by intravenous injection every other day to treat IBD mice until Day 13 [28, 29, 30]. In terms of weight recovery, OPR had the most significant effect, restoring the initial body weight of IBD mice from 66.8 ± 2.2% to 95.4 ± 2.1% (Figure 2G), which was better than 5‐ASA (85.3 ± 2.7%), DEX (82.8 ± 4.3%), UPA (88.1 ± 2.3%), and INF (91.1 ± 2.8%). However, GP (73.2 ± 2.6%) had no significant weight recovery effect due to its lack of intestinal targeting ability. In terms of DAI (Figure 2H), OPR also had a significant effect (reduce the DAI score of IBD mice from 3.8 ± 0.2 to 0.8 ± 0.3), which was better than 5‐ASA (2.0 ± 0.2), DEX (2.1 ± 0.2), UPA (1.2 ± 0.2), and INF (1.1 ± 0.3), while GP (3.3 ± 0.4) had no significant effect on the DAI. In terms of colon length (Figure 2I,J), OPR still had the most significant therapeutic effect, which could restore the colon length of IBD mice from 5.4 ± 0.5 cm to 7.6 ± 0.2 cm, close to the level of the normal group (8.2 ± 0.2 cm). The effect was better than 5‐ASA (6.5 ± 0.3 cm), DEX (6.5 ± 0.4 cm), UPA (6.9 ± 0.2 cm), and INF (7.0 ± 0.3 cm), while GP (5.7 ± 0.3 cm) failed to restore the colon length of mice. In terms of colon tissue structure and morphology (Figure 2K,L), H&E staining results of IBD group mice showed crypt loss, mucosal ulceration, infiltration of mixed inflammatory cells, and congestion of the muscularis propria. Treatment with OPR markedly reduced these pathological features, showing minimal ulceration, significantly reduced inflammatory infiltrate, and restored crypt architecture, effectively improving the intestinal damage and restoring barrier integrity. OPR reduced the intestinal epithelial injury score of IBD mice from 5.7 ± 0.3 to 0.8 ± 0.2, which was basically equivalent to that of the normal group (0.4 ± 0.2), and the effect was better than that of other positive drug treatment groups 5‐ASA (2.5 ± 0.2), DEX (2.6 ± 0.3), UPA (1.7 ± 0.2), and INF (1.6 ± 0.3). In summary, OPR showed superior treatment efficacy for IBD comparing to first‐line clinical drugs (5‐ASA, DEX, UPA, and INF), while GP exhibited minimal therapeutic effects in vivo due to its lack of intestinal targeting ability.

### OPR inhibition of *Clostridium difficile* toxicity

Given that IBD is closely related to intestinal flora imbalance, the effect of OPR on IBD intestinal flora was subsequently explored. We analyzed the effect of OPR on the flora of IBD mice (the OPR group) by 16S‐rRNA sequencing upon mouse feces, and healthy mice (the control group) and IBD mice (the IBD group) were used as controls. The chao1 index was used to assess the α diversity of the dataset. The IBD group was significantly lower than that of the control group, indicating that the species composition and diversity of the IBD group were significantly lower than that of the control group. The species abundance and diversity were close to those of control group after OPR treatment (Figure 3A). β diversity was assessed by the principal coordinate analysis (PCoA), which showed that the composition of the gut flora of the OPR group was significantly different from that of the IBD group and close to that of the control group (Figure 3B). The relative abundance of gut microbiota at the phylum level is shown in Figure 3C, and the gut microbiota was mainly distributed into *Proteobacteria*, *Firmicutes*, *Campylobacterota*, *Bacteroidota*, *Actinobacteriota*, and *Verrucomicrobiota* at the phylum level. Importantly, the *Clostridia* (belonging to *the Firmicutes* phylum) abundance increased in IBD mice at class level, and decreased significantly after OPR treatment. The 25 orders with the highest abundance value were further analyzed at the order level. Again, the abundance of the *Peptostreptococcales* order (belongs to *Clostridia* class) showed the most significant changes among the control group, IBD group, and OPR group (Figure 3D). Consistent with the order level data, the abundance of the *Peptostreptococcaceae* family (belonging to *Peptostreptococcales* order) significantly increased in IBD group (from 0.006 ± 0.002 to 0.337 ± 0.131), and OPR can decrease it to 0.020 ± 0.006 (Figure 3E). Finally, the abundance of *Clostridium difficile* (which belongs to the *Peptostreptococcaceae* family) was confirmed by qPCR. As expected, *Clostridium difficile* significantly increased in the IBD group (5.6 ± 1.5 folds of the control group), and OPR decreased it to 1.6 ± 0.2 folds (Figure 3F). These results indicate that OPR can recover intestinal flora balance and has a regulation effect on *Clostridium difficile*.

**Figure 3:**
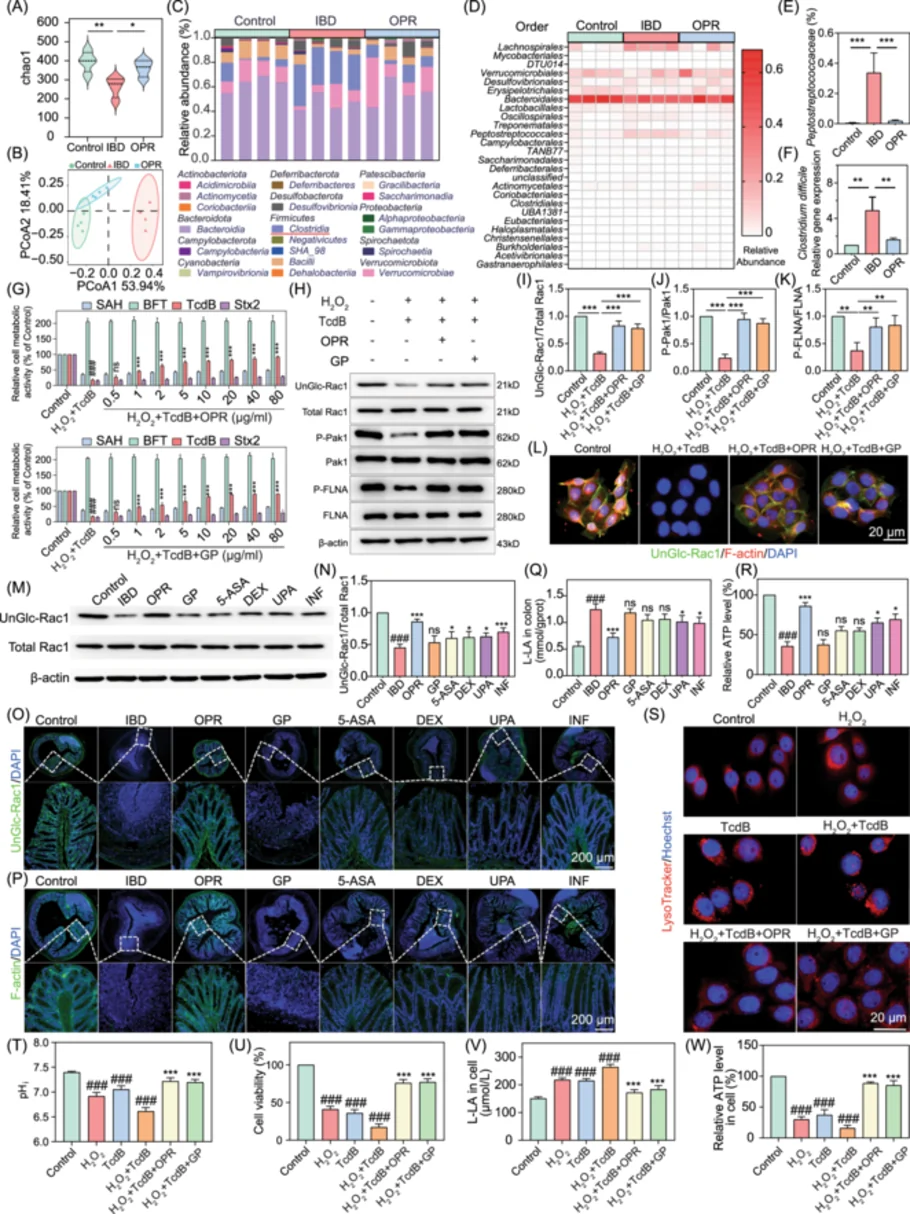
Analysis of intestinal microbiota in IBD mice and exploration of the mechanism of OPR inhibition of *Clostridium difficile* toxicity. (A) Chao1 index illustrating the gut microbiome α‐diversity among groups (*n* = 4). (B) PCoA plot illustrating the gut microbiome β‐diversity among groups (*n* = 4). (C) Bar chart of the relative abundance (phylum and class level) of gut microbiota in different groups (*n* = 4). (D) Heat map of the relative abundance of the top 25 order‐level gut microbiota in different groups (*n* = 4). (E) Relative abundance of *Peptostreptococcaceae* family in different groups (*n* = 4). (F) The relative expression level of *Clostridium difficile* among different groups was analyzed by qPCR (*n* = 3). (G) Therapeutic effects of different concentrations of OPR and GP on toxins‐affected HT29 cell metabolic activity were assessed by CCK‐8 assay (*n* = 3). (H) Western blot analysis of the protein expression related to Cytoskeleton in HT29 cells with different treatments and corresponding quantification (I–K) (*n* = 3). (L) Representative immunofluorescent staining images of HT29 cells after different treatments were stained with DAPI (blue), F‐actin (green), and Unglucosylated Rac1 (red). Scale bar is 20 µm (*n* = 3). (M) Western blot analysis of Unglucosylated Rac1 and Total Rac1 proteins expression in Mouse colon tissue in different groups and corresponding quantification (N) (*n* = 3). (O) Representative immunofluorescent staining images of UnGlc‐Rac1 protein of colon tissue from different groups. Scale bar is 200 µm (*n* = 3). (P) Representative immunofluorescent staining images of F‐actin of colon tissue from different groups. Scale bar is 200 µm (*n* = 3). (Q) L‐LA levels in mouse colon among different groups (*n* = 3). (R) ATP levels in mouse colon among different groups (*n* = 3). (S) Representative immunofluorescence staining of LysoTracker in HT29 cells under different treatments. Scale bar is 20 µm (*n* = 3). (T) Intracellular pH (pH_i_) in HT29 cells under different treatments (*n* = 3). (U) Cell viability of HT29 cells under different treatments (*n* = 3). (V, W) L‐LA (V) and ATP (W) levels in HT29 cells from different groups (*n* = 3). Data represent means ± S.D. Statistical significance was calculated by one‐way ANOVA with Tukey's post‐test, *p < 0.05, **p < 0.01, ***p < 0.001. For (G), ^###^ *p* < 0.001 vs. Control; **p < 0.01, ***p < 0.001 vs. H_2_O_2_ + Toxin. (ns: no statistical difference compared to H_2_O_2_ + Toxin). For (N), (Q), and (R), ^###^ *p* < 0.001 vs. Control; *p < 0.05, ***p < 0.001 vs. IBD. (ns: no statistical difference compared to IBD). For (T–W), ^###^ *p* < 0.001 vs. Control; ***p < 0.001 vs. H_2_O_2_ + TcdB. PCoA, principal co‐ordinates analysis; qPCR, quantitative polymerase chain reaction; DAPI, 4',6‐diamidino‐2‐phenylindole; L‐LA, L‐lactic acid.

To investigate the mechanism of OPR regulation on *Clostridium difficile*, we treated H_2_O_2_‐induced HT29 cells with TcdB toxin from *Clostridium difficile*, and evaluated whether OPR or GP could protect the IECs from the toxins by CCK‐8 assay. Three other IBD‐related bacterial toxins: *Bacteroides fragilis* toxin (BFT), *Staphylococcus aureus* α‐Hemolysin (SAH), and *Shiga* toxin 2 (Stx2) were used to confirm if the therapeutic effect of OPR is *Clostridium difficile* specific. Both OPR and GP inhibited the decrease in cell metabolic activity induced by TcdB in a dose‐dependent manner, whereas for other toxins (BFT, SAH, Stx2), OPR and GP could not well counteract the toxic effects brought about by these toxins (Figure 3G). The inhibition effects of OPR and GP on TcdB toxin in HT29 cells were quantified through CellTiter‐Glo® Luminescent Cell Viability Assay. The inhibition curves of OPR and GP on H_2_O_2_ + TcdB‐induced toxicity showed that both OPR and GP had excellent concentration‐dependent anti‐toxicity effects, with IC50 of 2.90 μg/mL and 2.45 μg/mL, respectively (Figure S12).

TcdB, as a glucosyltransferase, enters the cytosol via host cell receptor‐mediated endocytosis and specifically catalyzes glycosylation modification of Rho‐family GTPases such as Ras‐related C3 botulinum toxin substrate 1 (Rac1) [5]. This modification deprives Rac1 of the ability to phosphorylate the downstream effector protein P21‐activated kinase 1 (Pak1), which in turn blocks the activation cascade of Pak1 on cytoskeletal regulatory proteins (actin‐binding protein filamin, LIM kinase, myosin) [31]. Among them, the inhibition of filamin A (FLNA), as the core protein of F‐actin cross‐linking, directly led to the disintegration of cytoskeletal network [32]. To investigate the protective effect of OPR on cytoskeleton of IECs, we established a H_2_O_2_ + TcdB‐induced HT29 cell model. Through WB, Unglycosylated‐Rac1 (UnGlc‐Rac1) was reduced by 68% in HT29 cells under the effect of H_2_O_2_ + TcdB, and its downstream phosphorylation levels of Pak1 and FLNA decreased by 77% and 63%. The levels of non‐glycosylated Rac1 increased by 50% and 46% compared to the H_2_O_2_ + TcdB group after OPR or GP treatment (Figure 3H–K), respectively. The levels of Pak1 and FLNA phosphorylation downstream of Rac1 were increased by 70% and 43% or 63% and 47% after OPR or GP treatment, respectively. Meanwhile co‐staining of F‐actin and UnGlc‐Rac1 in HT29 cells showed that TcdB induction resulted in a decrease in UnGlc‐Rac1 levels in HT29 cells, accompanied by cytoskeletal cleavage (Figure 3L). In contrast, OPR or GP treatment significantly elevated UnGlc‐Rac1 levels and restored cytoskeletal morphology to a level close to that of the normal group. In vivo, UnGlc‐Rac1 levels were significantly decreased in the IBD group, and only the OPR group was able to reverse this process through WB (Figure 3M,N) and immunofluorescence (Figure 3O). Through immunofluorescence of F‐actin, the IBD group was accompanied by severe cytoskeleton disintegration, whereas cytoskeleton of the OPR‐treated IBD mice was close to those of the control group, which was significantly better than that of the other treatments (Figure 3P).

More importantly, the colon tissue in the IBD group was accompanied by significantly elevated levels of L‐LA (Figure 3Q) and decreased ATP levels (Figure 3R), and both L‐LA and ATP levels returned to normal after OPR treatment. We assumed that the mitochondria metabolism of IECs shifted from oxidative phosphorylation to glycolysis in IBD, and the increase of glycolysis led to the accumulation of L‐LA and a decrease in cytosolic pH. To verify if OPR could regulate mitochondria metabolism of IECs in IBD, we first used Lysotracker, a probe that targets acidic bodies in live cells, to assess HT29 cells in different treatment groups. The red fluorescence of the H_2_O_2_ group was significantly enhanced compared to control group, indicating that oxidative stress would lead to an increase of acidic bodies. In addition, cells in the H_2_O_2_ + TcdB group showed worse cell morphology and increased acidic bodies compared to the TcdB group, indicating that TcdB could exert a more severe toxic effect under acidic environment. The cell morphology recovered, and the number of acidic bodies decreased significantly after OPR and GP treatment, indicating that OPR and GP could counteract the toxic effects of H_2_O_2_ + TcdB in IECs (Figure 3S). To quantify the ability of OPR and GP to regulate cytoplasmic pH in IECs, we used pH probes to measure intracellular pH (pH_i_). The results showed that the pH of the H_2_O_2_ group decreased from 7.40 ± 0.02 to 6.92 ± 0.06, which manifested as cytosolic acidification. The TcdB group also showed a slight decrease in pH_i_. In addition, the pH_i_ of the H_2_O_2_ + TcdB group (6.61 ± 0.06) was lower than that of H_2_O_2_ group (6.92 ± 0.06) and TcdB group (7.06 ± 0.06). The pH_i_ of the OPR group and the GP group recovered to 7.22 ± 0.06 and 7.20 ± 0.05, respectively, which was close to that of the control group (Figure 3T). The cell viability assay showed similar trends as the pH_i_ results: the lower pH_i_, the lower cell viability (Figure 3U). These results suggest that OPR and GP can inhibit the toxic effects of TcdB by restoring the cytoplasmic pH of damaged (oxidative‐stressed) IECs. To verify if L‐LA was the key factor that caused cytoplasmic pH decline, we further tested the L‐LA levels in different groups, and the results showed that the L‐LA levels in each group corresponded to the pH_i_ values. The L‐LA content in control group was 151 ± 5 μmol/L, and the L‐LA content in the H_2_O_2_ + TcdB group was 264 ± 8 μmol/L, while the L‐LA level was recovered to 172 ± 9 μmol/L and 184 ± 11 μmol/L after OPR and GP treatment (Figure 3V). Finally, we measured the ATP level in the cells, and the results showed that the ATP level in the H_2_O_2_ + TcdB group significantly decreased, which was only 16% of the control group, and recovered to 89% and 85% after OPR and GP treatment, respectively (Figure 3W). In order to confirm that the efficacy of OPR in reducing cellular L‐LA accumulation and restoring cellular ATP synthesis is due to the regulation of cell metabolic reprogramming, we supplemented oxygen consumption rate (OCR) and extracellular acidification rate (ECAR) assays in the HT29 cell model, and the results showed that the OCR of cells in the model group was significantly reduced, and the ECAR was significantly increased, while OPR/GP could significantly restore the above indicators (Figure S13). The results of OCR and ECAR tests fully proved that OPR could restore cell oxidative phosphorylation, inhibit cellular glycolysis, and regulate cell metabolic reprogramming. We also measured the ratio of oxidized/reduced nicotinamide adenine dinucleotide (NAD^+^/NADH) and ATP/ADP in mouse colon samples, and analyzed the level/activity of hexokinase 2 and 6‐phosphate fructokinase (PFK) in the glycolytic pathway. The results showed that the proportion of NAD^+^/NADH decreased, the ratio of ATP/ADP decreased, the activity of hexokinase 2 increased, and the activity of fructokinase 6‐phosphate increased in the IBD‐CDI model group. OPR showed a more significant recovering effect on the above indicators comparing to other drugs (Figure S14). These results further confirm that OPR plays a therapeutic role by regulating the glycolysis process of IECs.

In summary, we speculated that OPR could restore MMP by inhibiting UCP2, reprogram the metabolism of IECs from glycolysis to oxidative phosphorylation, reduce the production of L‐LA, inhibit the formation of acidic vesicles, thereby alleviating the toxic effect of TcdB.

### Therapeutic effect of OPR in CDI‐complicated IBD

To further confirm the effect of OPR on inhibiting TcdB toxicity, we established an IBD mouse model complicated by CDI. The mice were fed with 3% DSS for seven consecutive days to destroy the intestinal barrier of mice and inoculated 10^6^ colony‐forming units (CFU) of toxigenic *Clostridium difficile* by gavage on the 7th day to construct an IBD‐CDI mouse model (Figure 4A). OPR (5 mg/kg) was administered orally on Days 7, 9, and 11 to treat the IBD‐CDI mice. A first‐line combination medication group was set up to compare efficacy with OPR: Fidaxomicin‐Infliximab (Group F‐I) [4, 33]. Group F‐I received Fidaxomicin (30 mg/kg) orally on Days 7–9 and Infliximab (5 mg/kg) intravenously on Days 10–12 [34]. Given that the IBD‐CDI mouse model has a higher degree of intestinal damage and a more complex pathological mechanism, the therapeutic effect of OPR in the IBD‐CDI model was evaluated with selected indicators such as mortality, body weight, DAI, intestinal epithelial injury score, colon length, bacterial load in intestinal contents after *Clostridium difficile* inoculation, and TcdB content. We observed that the IBD + CDI group (58.0%) had a higher mortality rate than the IBD group (25.0%), suggesting that *Clostridium difficile* colonization and infection can aggravate IBD symptoms. The OPR group could significantly reduce the mortality of IBD + CDI mice (12.5%), and the effect was better than that of the F‐I group (33.0%) (Figure 4B). OPR significantly restored the body weight of IBD + CDI mice from the initial value of 72.7 ± 2.9% to 98.2 ± 1.5%, and the effect was better than that in the F‐I group (90.3 ± 3.0%) (Figure 4C). OPR was also effective, reducing the DAI of IBD + CDI mice from 3.7 ± 0.3 to 0.9 ± 0.4, and the effect was significantly better than that in the F‐I group (2.0 ± 0.5) (Figure 4D). Through H&E staining, the colons of mice in the IBD and IBD + CDI groups were accompanied by obvious crypt loss, mucosal ulceration, infiltration of mixed inflammatory cells and congestion of the muscularis propria. OPR could significantly alleviate the above pathological changes, and the effect was better than that of the F‐I group (Figure 4E). The intestinal epithelial injury score results showed that the intestinal epithelial injury in the IBD + CDI group (5.7 ± 0.3) was more severe than that in the IBD group (5.1 ± 0.4). OPR could reduce the intestinal epithelial injury score of IBD + CDI mice from 5.7 ± 0.3 to 1.2 ± 0.3, and the effect was better than that in the F‐I group (2.5 ± 0.5) (Figure 4F). In addition, the colon of the IBD + CDI group (4.8 ± 0.2 cm) was shorter than that of the IBD group (5.5 ± 0.1 cm). OPR could restore the colon length of IBD + CDI mice to 7.0 ± 0.3 cm, approaching the level of the normal group (7.6 ± 0.2 cm), and the effect was better than that of the F‐I group (5.4 ± 0.4 cm) (Figure 4G,H). Interestingly, the bacterial load of *Clostridium difficile* and the content of TcdB in the IBD + CDI + OPR group was significantly reduced compared with the IBD + CDI group, the decrease was not large, only 27.0% and 25.8%, which was lower than the antibacterial effect of the F‐I group (48.5% and 50.3%) (Figure 4I,J), indicating that OPR does not treat CDI by directly killing/inhibiting *Clostridium difficile* or reduce TcdB release through antibiotic‐like effects. To verify if OPR treated IBD‐CDI by inhibiting the toxicity of TcdB in IECs, we detected the cytoskeleton‐related proteins through WB. The non‐glycosylated proportion of Rac1 in the IBD + CDI group (32.2 ± 3.8% of the control group) was lower than that in the IBD group (46.1 ± 6.4% of the control group). OPR could effectively reduce the glycosylation level of Rac1, and restore the non‐glycosylated proportion of Rac1 in the IBD + CDI and IBD groups, which was much better than that in the F‐I group (Figure 4K–N). In addition, OPR could significantly restore the levels of intestinal barrier tight junction proteins in the IBD + CDI group, such as E‐cadherin and Occludin, which was better than that in the F‐I group. By ELISA, OPR can significantly reduce the inflammatory factors (tumor necrosis factor‐alpha (TNF‐α), interleukin 1 beta (IL‐1β), interleukin 6 (IL‐6)) in the colon tissue of IBD + CDI mice, and the effect is significantly better than that of the F‐I group (Figure 4O–Q).

**Figure 4:**
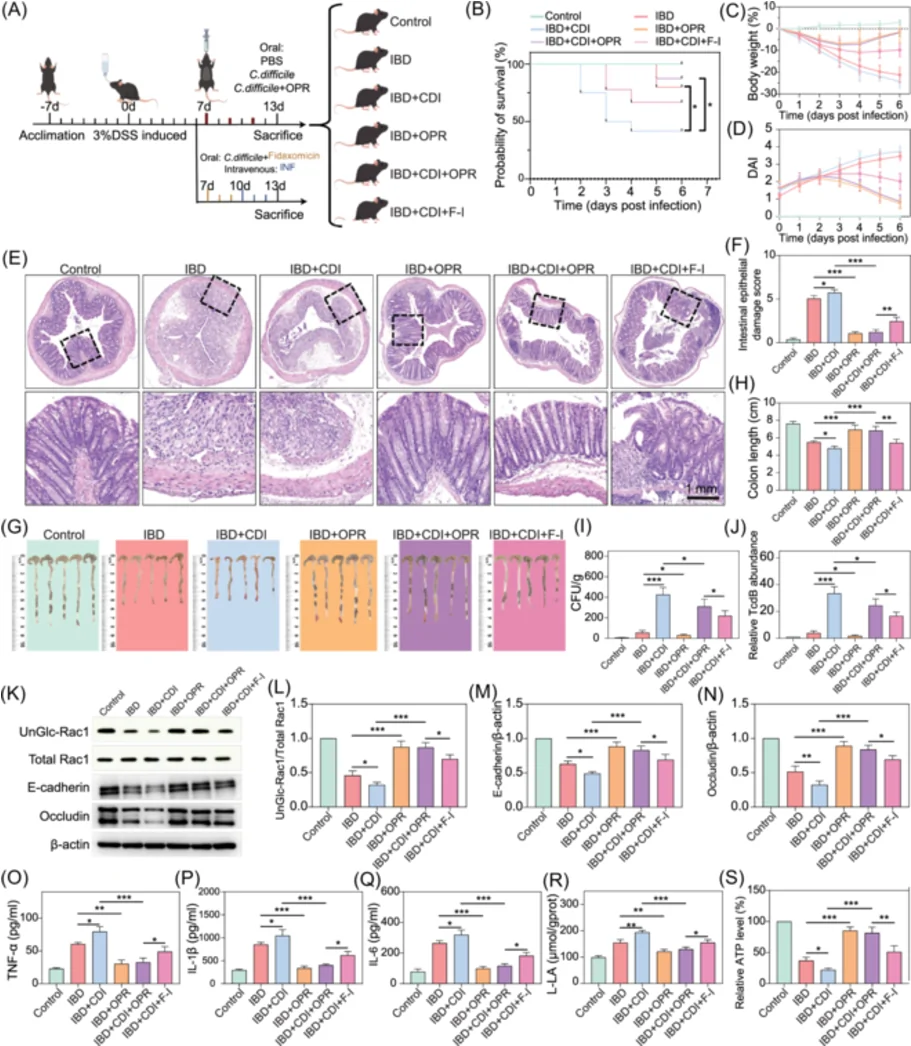
Therapeutic effect of oral proton‐reprogrammed (OPR) in inflammatory bowel disease‐clostridium difficile infection (IBD‐CDI) model. (A) Diagram of IBD‐CDI model construction. (B) Survival analysis of the mice post CDI (*n* = 8–12). (C) Weight change of the mice post CDI (*n* = 5). (D) DAI analysis of the mice post CDI (*n* = 5). (E) H&E staining of intestinal sections from different groups. Scale bar is 1 mm (*n* = 5). (F) Epithelial injury score evaluation among different groups (*n* = 5). (G) Colon length comparison among different groups (*n* = 5). (H) Gross specimen of colon length comparison among different groups (*n* = 5). (I) *Clostridium difficile* burden in intestinal contents in different groups (*n* = 5). (J) qPCR detection of TcdB content in intestinal contents in different groups (*n* = 5). (K–N) Western blot detection and quantification of UnGlc‐Rac1 (L), E‐cadherin (M), Occludin (N) (*n* = 3). Inflammatory cytokine levels in colon among different groups: TNF‐α (O), IL‐1β (P), IL‐6 (Q) (*n* = 3). L‐LA (R) and ATP (S) levels in mouse colon from different groups (*n* = 3). Data represent means ± S.D. Statistical significance was calculated by one‐way ANOVA with Tukey's post‐test, *p < 0.05, **p < 0.01, ***p < 0.001. CDI, *Clostridium difficile* infection; TNF‐α, tumor necrosis factor‐alpha; IL‐1β, interleukin 1 beta; IL‐6, interleukin 6.

In IBD, a sharp increase of ROS can induce UCP2 over‐activation and damage the structural integrity of mitochondria of IECs, resulting in the inability to carry out oxidative phosphorylation, forcing cells to undergo glycolysis, which increased L‐LA content and decreased pH in the colon of IBD patients [35]. The acidification of IECs provides a suitable environment for *Clostridium difficile* to exert its toxic effects, forming an IBD‐CDI vicious cycle. As expected, the L‐LA level in the IBD + CDI group (1.38 ± 0.04 mmol/gprot) was higher than that in the IBD group (1.16 ± 0.04 mmol/gprot). OPR could reduce the L‐LA level in the IBD + CDI group to 0.78 ± 0.05 mmol/gprot, which was significantly better than that in the F‐I group (0.93 ± 0.06 mmol/gprot) (Figure 4R). The ATP level in the IBD + CDI group (22.0 ± 2.6%) was lower than that in the IBD group (37.0 ± 5.3%). OPR could restore the ATP level in the IBD + CDI group to 81.7 ± 9.3%, which was close to the level of the control group (100%) and was significantly better than that in the F‐I group (51.0 ± 9.8%) (Figure 4S).

The results above suggest that OPR plays a role in restoring the intestinal barrier and inhibiting the glycosylation of Rac1 induced by TcdB in the IBD + CDI model, and its therapeutic effect is better than the complicated treatment of fidaxomicin and infliximab. OPR can restore mitochondrial function of IECs, and effectively inhibit cellular acidification caused by mitochondrial reprogramming, thereby resisting the toxic effects of *Clostridium difficile*.

### OPR can recover mitochondrial function and inhibit the GSDME‐mediated pyroptosis of IECs to restore the intestinal barrier

Mitochondria of IECs are key for the treatment in both IBD and CDI‐complicated IBD. Given that the mechanism of OPR in resisting the damage of TcdB has been fully verified in the above sections, in the following experiments, we focus on the role of OPR in repairing the mitochondrial function of IECs in IBD. Firstly, we used tetramethylrhodamine ethyl ester (TMRE) as a probe at the in vitro level (simulating the pH environment in IBD lesions) to detect the effects of OPR sustained‐release products at different time points (the longer the release time, the higher the GP content) on MMP in real time, and to determine the recovery of mitochondrial function by detecting ATP content. In the preliminary experiment, we set up five OPR concentration gradients (1, 3, 10, 30, 100 μg/mL), and finally selected OPR (10 μg/mL) as the cell experimental concentration based on the results of CCK‐8 cell viability assay (Figure S15) and cellular ROS detection (Figure S16). The MMP dropped to 59.6% of the normal MMP after 15 min of induction with 2 mM H_2_O_2_, and GP was able to restore the MMP to about 85% of the normal MMP within 15 min. Interestingly, the longer the in vitro sustained‐release time of the OPR product, the greater its ability to restore MMP. The OPR product collected from a sustained release for 168 h can restore the MMP to 82% of the initial value within 15 min (Figure 5A,B). Subsequently, the addition of a potent mitochondrial uncoupler, carbonyl cyanide 3‐chlorophenylhydrazone, could immediately and significantly reduce MMP in the GP or OPR groups. We simultaneously detected the ATP content in different treatment groups at the 30‐min time point. Consistent with the results of MMP, GP could restore the cellular ATP content to 1.93 times that of the H_2_O_2_ group. The longer the in vitro sustained release time of the OPR product, the stronger the ATP recovery ability. The OPR product after a sustained release for 168 h in vitro could restore the cellular ATP content to 1.78 times that of the H_2_O_2_ group (Figure 5C). The above results indicate that OPR can restore MMP by releasing GP and inhibiting UCP2, thereby restoring mitochondrial function.

**Figure 5:**
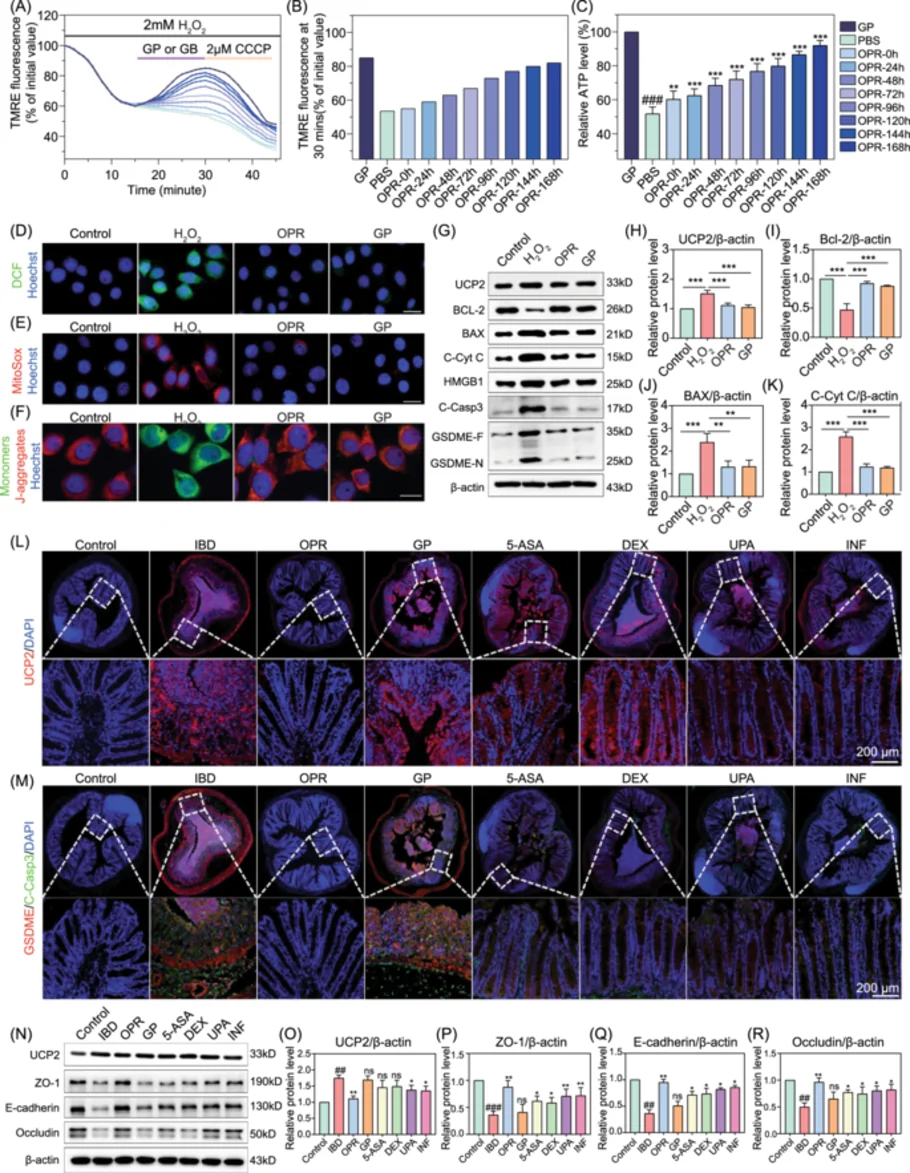
Oral proton‐reprogrammed (OPR) alleviates oxidative stress, repairs mitochondrial function, and restores the intestinal barrier. (A) Real‐time MMP levels indicated by TMRE fluorescence in HT29 cells. The straight line above the image indicates the different stages of processing. (B) Relative fluorescence intensity of TMRE in each group 10 min after OPR or GP treatment. (C) ATP content of HT29 cells treated with OPR or GP (*n* = 3). (D) Representative image of immunofluorescence staining of DCFH‐DA in HT29 cells under different treatments. Scale bar is 20 µm (*n* = 3). (E) Representative image of immunofluorescence staining of MitoSox in HT29 cells under different treatments. Scale bar is 20 µm (*n* = 3). (F) Representative image of immunofluorescence staining of JC‐1 in HT29 cells under different treatments. Scale bar is 10 µm (*n* = 3). (G–K) Western blot analysis of UCP2 (H), BCL‐2 (I), BAX (J), and C‐Cyt C (K) proteins in HT29 cells of different groups and corresponding quantification (*n* = 3). Representative immunofluorescent staining images of UCP2 (L) and GSDME (M) proteins in colon tissue from different groups. Scale bar is 200 µm (*n* = 3). (N–R) Western blot analysis of UCP2 (O), ZO‐1 (P), E‐cadherin (Q), and Occludin (R) proteins in colon tissue from different groups and corresponding quantification (*n* = 3). Data represent means ± S.D. Statistical significance was calculated by one‐way ANOVA with Tukey's post‐test. ^##^ *p* < 0.01, ^###^ *p* < 0.001 vs. Control; *p < 0.05, **p < 0.01,***p < 0.001 vs. IBD. (ns: no statistical difference compared to IBD). For (C), ^###^ *p* < 0.001 vs. GP, **p < 0.05, ***p < 0.001 vs. PBS. For (H–K), **p < 0.01, ***p < 0.001. MMP, mitochondrial membrane potential; TMRE, tetramethylrhodamine ethyl ester; DCFH‐DA, 2',7'‐dichlorodihydrofluorescein diacetate; BCL‐2, B‐cell lymphoma 2; BAX, BCL2‐associated X; C‐Cyt C, cytosolic cytochrome complex.

Considering that the inhibition of UCP2 may increase ROS levels, the ability of OPR to scavenge ROS is crucial for its therapeutic role. We used the 2',7'‐dichlorodihydrofluorescein diacetate (DCFH‐DA) probe to detect the ROS level in HT29 cells induced by H_2_O_2_ and examined the intracellular ROS scavenging ability of OPR. Immunofluorescent (Figure 5D) and flow cytometry (Figure S17A) results showed that OPR and GP could reduce the ROS level in HT29 cells to near normal levels, indicating that both OPR and GP have excellent intracellular ROS scavenging effects. Mitochondria are an important site to produce ROS in cells. With MitoSox fluorescent staining, OPR could reduce the over‐elevated mitochondria ROS (mtROS) levels induced by H_2_O_2_ in HT29 cells (Figure 5E, Figure S17B). Through JC‐1 fluorescent staining (Figure 5F) and flow cytometry (Figure S17C), OPR and GP were as effective in restoring MMP in HT29 cells induced by H_2_O_2_. OPR's potent mitochondrial repair effect is not only related to its ability to efficiently eliminate ROS and inhibit UCP2, but also to its ability to highly target mitochondria. Next, we incubated Mitotracker (a mitochondria dye) and OPR‐FITC with HT29 cells to observe the ability of OPR to target mitochondria in cells. As shown in Figure S18, Mitotracker and OPR‐FITC were highly co‐localized, and their Pearson's coefficient was 0.83, indicating that OPR has excellent mitochondrial targeting. Mitochondrial targeting groups (like Triphenyl phosphate (TPP)) are usually groups with strong positive charges, which are mainly achieved by targeting the negatively charged mitochondrial phospholipid‐cardiolipin. Given that the surface of OPR has a strong negative charge, we speculate that OPR is targeted to mitochondria mainly through mitochondrial membrane proteins rather than mitochondrial phospholipids. We further explored the binding ability of OPR, GP and mitochondrial outer membrane proteins through molecular docking. The binding energies of OPR to mitochondrial outer membrane proteins (translocase of outer mitochondrial membrane 20 (TOM20), translocase of outer mitochondrial membrane 34 (TOM34), voltage‐dependent anion channel 1 (VDAC1), voltage‐dependent anion channel 2 (VDAC2), translocase of outer mitochondrial membrane 70 (TOM70), and heat‐shock protein 90 (HSP90)) were −24 kcal/mol, −28.8 kcal/mol, −19.4 kcal/mol, −12.9 kcal/mol, −10.5 kcal/mol, and −15.5 kcal/mol respectively, obviously higher than GP's −3.3 kcal/mol, −3.5 kcal/mol, −3.2 kcal/mol, −4.7 kcal/mol, −4.2 kcal/mol, and −3.3 kcal/mol (Figure S19A–D). Therefore, OPR is more likely to target the mitochondrial outer membrane, and OPR releases UCP2‐specific inhibitor (GP) to exert its effect after reaching mitochondria. Malondialdehyde (MDA) is a key indicator of the oxidative stress degree in IBD because excessive ROS can oxidize lipids and produce high concentrations of MDA in IBD lesions. As shown in Figure S19E, the intestinal MDA level of IBD mice increased significantly, and OPR could restore MDA to a level close to that of the control group, which was better than 5‐ASA, DEX, UPA, and INF. GP had no significant ability to regulate intestinal MDA. To confirm whether OPR exerts mitochondrial protection by regulating UCP2, we constructed a UCP2 overexpression plasmid (UCP2‐OE) and transfected it into HT29 cells. We first verified that the transfection efficiency of UCP2‐OE in HT29 cells was about 82% (Figure S20A), and then confirmed that the UCP2 overexpressing HT29 cell line was successfully constructed by qPCR and WB (Figure S20B,C). To verify whether the therapeutic effect of OPR is based on specific inhibition of UCP2, DCF, JC‐1, and MitoSOX staining were performed in HT29 cells overexpressing UCP2 (Figure S21A,B). Unlike H_2_O_2_ stimulation, UCP2‐OE does not cause an increase in ROS, mtROS (which should theoretically decrease); therefore, there was no significant difference between the UCP2‐OE group and the Control group in DCF and MitoSOX detection. Similar to H_2_O_2_ stimulation, UCP2‐OE can significantly reduce mitochondrial membrane potential, so the UCP2‐OE group is significantly different from the Control group in the detection of cell JC‐1, and OPR and GP can restore the mitochondrial membrane potential. The above results fully prove that the mitochondrial protection of OPR is exerted by targeted regulation of UCP2.

To confirm the ROS scavenging ability of OPR in vivo, we detected the ROS level in mouse intestinal tissue. As shown in Figure S22, OPR can significantly eliminate the abnormally elevated ROS in the intestines of IBD mice, and its effect is better than 5‐ASA, DEX, UPA, and INF. GP cannot eliminate ROS in the intestine of mice due to its lack of intestinal targeting ability. In summary, OPR not only restores mitochondrial function by inhibiting UCP2 but also targets mitochondria and scavenges mtROS, thereby significantly reducing oxidative stress in IBD.

Excessive ROS directly damages the IECs and leads to pyroptosis of IECs through the mitochondrial pathway, and ultimately leads to the disruption of the integrity of the intestinal barrier function [36]. To investigate the effect and mechanism of OPR in intestinal barrier restore, we observed DNA strand breaks (a marker of cellular pyroptosis) by terminal deoxynucleotidyl transferase dUTP nick end labeling (TUNEL) staining of mouse colon tissues. Obvious DNA strand breaks were observed in the IBD group, and the intensity of TUNEL staining was significantly elevated, which was 3.66 times higher than that of the control group. OPR can reduce the TUNEL fluorescence intensity of intestinal tissue of IBD mice to 1.12 times that of the normal group, and the effect is significantly better than that of other treatment groups (Figure S23A,B), suggesting that OPR plays a role in protecting cells from death at the tissue level. *In vitro*, we performed Live/Dead cell staining on H_2_O_2_‐induced HT29 cells with Calcein‐AM and Propidium Iodide (PI). As shown in Figure S23C, OPR and GP were able to protect HT29 cells from H_2_O_2_‐induced cell death. We also performed flow cytometry experiments on different treated HT29 cells using AnnexinV‐PI staining. The results showed that cell death in the OPR and GP treatment groups decreased from 35.1% to 13.2% and 14.2%, which was close to the normal level (9.79%) (Figure S23D).

Oxidative stress mediates pyroptosis of IECs through a mitochondria‐dependent pathway. In response to excess ROS, BAX is transported from the cytoplasm to the outer mitochondrial membrane, forming the permeability transition pore (MOMP), followed by the release of cytosolic cytochrome complex (C‐Cyt C) and ultimately activation of Caspase3, which triggers GSDME‐N‐mediated pyroptosis and the release of cellular contents such as mtDNA. To this end, we constructed an oxidative stress model using 2 mM H_2_O_2_‐induced HT29 and detected the indicators related to pyroptosis by WB. HT29 cells induced by H_2_O_2_ treated with OPR were able to effectively reduce UCP2, BAX, C‐Cyt C, C‐Casp3, and GSDME‐N, high‐mobility group box 1 (HMGB1) expression levels by 25%, 45%, 52%, 65%, 76%, and 48%, respectively, while BCL‐2 expression increased by 96% (Figure 5G–K, Figure S24). The effect of GP was similar to that of OPR. In vivo, the protein level of UCP2, BAX, C‐Cyt C, C‐Casp3, and GSDME was significantly upregulated in IBD mice, and was restored to the level close to that of the control group after OPR treatment (Figure 5L,M, Figures S25, S26A–D). Next, the intestinal repair by OPR was confirmed through immunofluorescence staining and WB on mouse colon tight junction proteins. The immunofluorescence staining (Figure S27A–C) showed that the OPR group was able to restore the intestinal tight junction proteins such as Occludin, ZO‐1, MUC2, and E‐cadherin to the level close to that of the control group. Whereas the other treatment groups, GP, 5‐ASA, DEX, UPA, and INF, showed only a slight improvement. Similarly, the WB results showed (Figure 5N) that the levels of tight junction proteins, such as ZO‐1, E‐cadherin, and Occludin, in the OPR group reached 88%, 95%, and 96% of those of the control group, respectively, which were significantly higher than those of other intervention groups (Figure 5P–R). Compared with other positive drugs, OPR showed a more significant regulatory effect on UCP2 in the colon tissues of IBD mice (Figure 5O). Taken together, the results suggest that OPR restores the intestinal barrier function through inhibiting excess ROS‐mediated pyroptosis and promoting the expression of tight junction proteins. More importantly, OPR shows better efficacy than other positive drugs due to its specific targeting of UCP2.

### OPR inhibits epithelial mtDNA‐mediated aberrant activation of the macrophage cGAS‐STING pathway

In addition to dominating intracellular energy metabolism, mitochondria are also involved in mediating inflammatory responses. In IBD, the aberrant opening of mitochondrial permeability transition pore (mPTP) in mitochondria of IECs leads to leakage of mtDNA into the cytoplasm, which triggers downstream inflammatory responses [37]. We first assessed the release of mtDNA in HT29 cells by dsDNA and TOM20 fluorescence co‐staining. As shown in Figure 6A,B, both OPR and GP significantly reduced mtDNA release induced by H_2_O_2_ treatment. In IBD, pyroptosis of IECs is closely related to subsequent inflammatory response [38]. The mtDNA leaked from pyroptotic IECs induce the activation of the cGAS‐STING pathway in macrophages to activate the inflammatory storm in IBD lesions. To investigate whether reducing IEC pyroptosis via OPR could subsequently attenuate macrophage activation, we co‐cultured HT29 cells in the upper chamber and the macrophage cell line RAW264.7 in the lower chamber in a Transwell system. The HT29 cells were first stimulated with H_2_O_2_ in the upper chamber to induce pyroptosis and intervened with the addition of OPR and GP, respectively, and the macrophages in the lower chamber were collected after 48 h (Figure 6C). Through WB, the H_2_O_2_ group induced abnormal activation of the cGAS‐STING pathway in macrophages, with increases of 105%, 66%, 122%, 98%, and 296% in cyclic GMP‐AMP synthase (cGAS), phosphor‐stimulator of interferon genes (P‐STING), stimulator of interferon genes (STING), phospho‐interferon regulatory factor 3 (P‐IRF3), and phospho‐nuclear factor kappa B‐P65 (P‐P65) compared to the control group, respectively, whereas decreases of 37%, 30%, 42%, 38% and 60% were observed in the OPR group compared to the IBD group (Figure 6D–I), indicating OPR were able to reduce the pyroptosis of HT29 cells, and attenuated cGAS‐STING pathway activation of macrophage.

**Figure 6:**
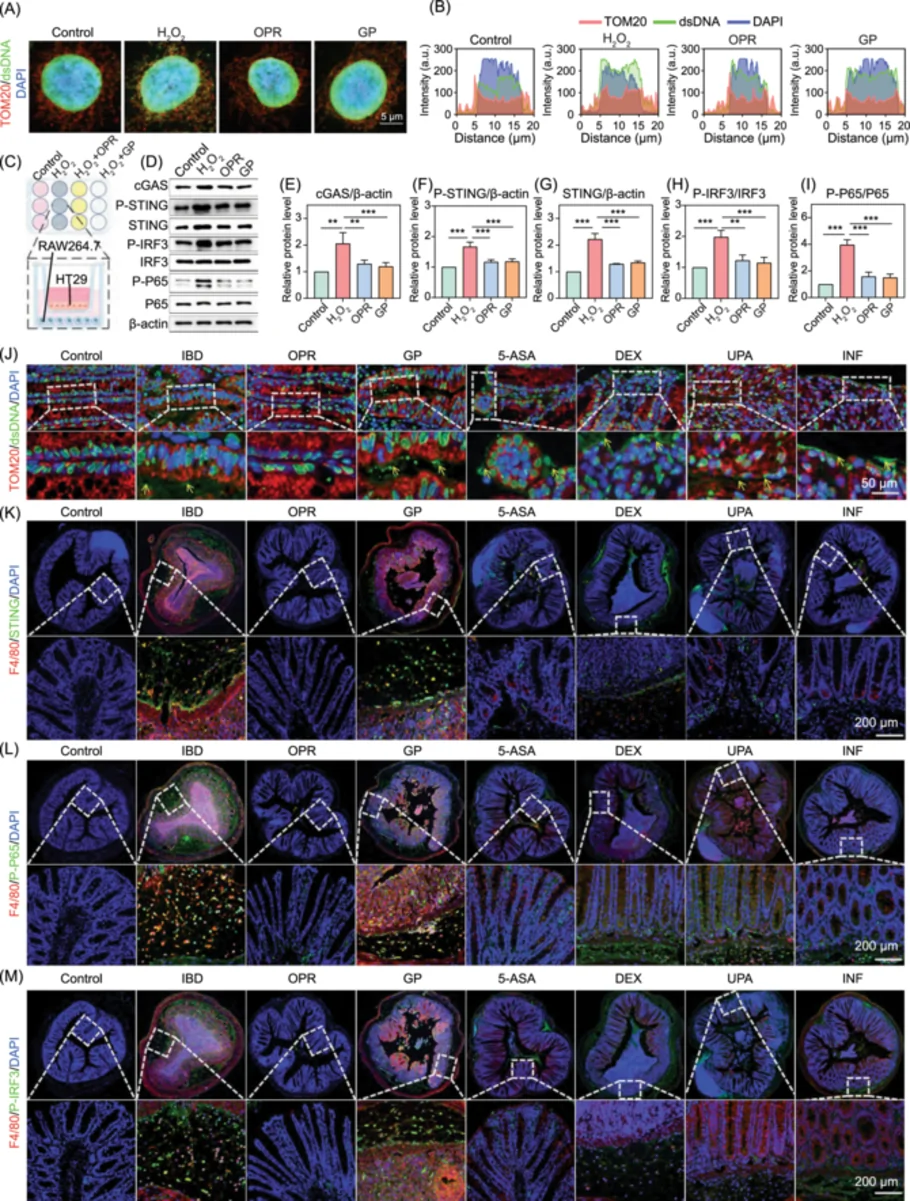
Oral proton‐reprogrammed (OPR) inhibits epithelial mtDNA‐mediated aberrant activation of the macrophage cGAS‐STING pathway. (A, B) Representative confocal fluorescence staining images of HT29 cells after different treatments were stained with DAPI (blue), dsDNA (green), and TOM20 (red) (A) and colocalization quantification (B). Scale bar is 5 µm (*n* = 3). (C) Schematic illustration of the HT29 cells and RAW264.7 cells co‐incubation experimental process. (D) Western blot analysis of the protein expression related to the cGAS‐STING pathway in RAW264.7 macrophages with different treatments and corresponding quantification (E–I) (*n* = 3). (J) Representative fluorescence staining images of different groups of colon tissues after different treatments were stained with DAPI (blue), dsDNA (green), and TOM20 (red). Yellow arrows indicate the released dsDNA. Scale bar is 50 µm (*n* = 3). Immunofluorescent staining images of F4/80‐STING (K), F4/80‐P‐P65 (L), and F4/80‐P‐IRF3 (M) in colon tissues from different groups. Scale bar is 200 µm (*n* = 3). Data represent means ± S.D. Statistical significance was calculated by one‐way ANOVA with Tukey's post‐test, **p < 0.01, ***p < 0.001. cGAS, cyclic GMP‐AMP synthase; STING, stimulator of interferon genes.

To demonstrate whether OPR could show the same effect in vivo, we assessed the effect of different groups of drugs in inhibiting mtDNA release from mitochondria by dsDNA and TOM20 fluorescence co‐staining in mouse colon tissues. Consistent with the results of cell staining, mtDNA diffusion was observed in both IBD and GP groups, and OPR was able to inhibit mtDNA release to a large extent; the effect of OPR was more significant than that of other positive drugs (Figure 6J, Figure S28). To better demonstrate that the activation of the STING pathway occurred predominantly within macrophages, we co‐stained mouse colon tissues with F4/80‐STING, F4/80‐P‐P65, and F4/80‐P‐IRF3, respectively. The F4/80^+^ macrophage infiltration in the mucosal and submucosal layers was significantly increased in the IBD group and was accompanied by high STING expression. Significant nuclear translocation of P‐P65 and P‐IRF3 was also observed in F4/80^+^ macrophages, which suggests the presence of activation of the STING pathway in macrophages in the colon of the IBD mouse. Only the OPR group was able to minimize the fluorescent signals of F4/80, STING, P‐P65, and P‐IRF3 to a level close to that of the control group, which was significantly better than that of the other intervention groups (Figure 6K–M). Finally, after OPR treatment, the elevated inflammatory factors TNF‐α (Figure S29A), IL‐1β (Figure S29B), and IL‐6 (Figure S29C) in the IBD group could be decreased by 50%, 66%, and 64%, respectively. The therapeutic effect of OPR was significantly superior to that of GP, 5‐ASA, DEX, UPA, and INF. Myeloperoxidase (MPO) reflects the infiltration of inflammatory cells in colon tissue and is also an important evaluation indicator of the severity of IBD. Consistent with the results of the inflammatory factors, OPR can restore the abnormally elevated MPO in the intestine of IBD mice to the level of the control group, and the effect is better than 5‐ASA, DEX, UPA, and INF (Figure S30). The above results proved that OPR could reduce the release of cellular contents (mtDNA), thereby inhibits macrophage STING pathway activation and significantly attenuate the subsequent inflammatory response.

### OPR treatment mechanism verified by single‐cell sequencing and transcriptomic analysis

Finally, we verified the therapeutic mechanism of OPR by single‐cell sequencing and transcriptome sequencing. A comprehensive analysis was performed with the human single‐cell sequencing dataset [GEO]GSE266616 from the GEO database. By combining automated annotation with manual annotation using well‐defined marker genes, we annotated 12 cell types across all lineages. We found that there was a significant decrease in IEC abundance as well as an increase in immune cell abundance (macrophages, NK cells, T cells) in patients with IBD (Figure 7A–C). We next analyzed the single‐cell sequencing results for DEGs. The results showed that epithelial cells, B cells, and macrophages had the highest number of DEGs, 677, 604, and 437, respectively (Figure 7D,E). Considering the number of DEGs and the correlation between cell types and IBD, we next focused on IECs and macrophages. In IECs of IBD patients, 460 DEGs were upregulated, and 183 DEGs were down‐regulated (Figure 7F). KEGG enrichment analysis of DEGs in epithelial cells revealed that they were significantly enriched in pathways involved in intercellular adhesion, inflammatory response, intestinal microorganisms, and ROS (Figure 7G). There were 357 DEGs upregulated and 24 DEGs downregulated in macrophages of IBD patients (Figure 7H). Enrichment analysis of KEGG, a DEG in macrophages, revealed that it was mainly associated with STING pathway, inflammatory response, and intestinal flora (Figure 7I). We further analyzed the expression of *UCP2*, *GSDME*, and *STING* in different cells of IBD patients and normal group, which was consistent with our conjecture: *UCP2*, *GSDME*, and *STING* were significantly increased in IBD patients (Figure S31A–C). *UCP2* was mainly expressed in the IECs (Figure S31A). *GSDME* was mainly expressed in the IECs (Figure S31B). *STING* was mainly expressed in the macrophages (Figure S31C). Once again, these single‐cell sequencing evidence confirmed that in human IBD, UCP2 expression in IECs increased, ROS levels increased, IECs pyroptosis occurred, and the cGAS‐STING pathway in macrophages was activated.

**Figure 7:**
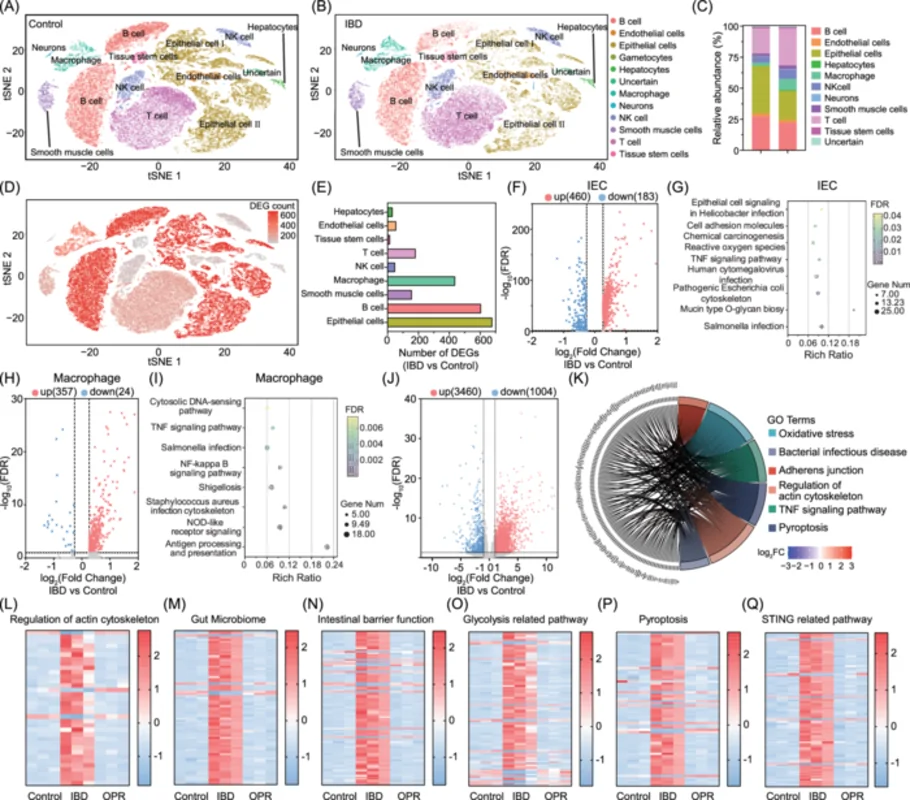
Single‐cell sequencing dataset of human colon and mouse colon transcriptomic sequencing analysis. (A–C) 12 cell types were identified in colonic mucosa tissue using graph‐based clustering by the t‐SNE (A, B) and the percentage of 12 cell types (C). (Control *n* = 13, IBD *n* = 13). (D) The t‐SNE plot and (E) bar chart show the number of DEGs across different cell types in the colon between Control group and IBD group. (Control *n* = 13, IBD *n* = 13). (F) Volcano map shows the number of DEGs in epithelial cells between control group and IBD group (|log_2_FC| > 0.25 and FDR < 0.05). (Control *n* = 13, IBD *n* = 13). (G) KEGG enrichment analysis of DEGs between Control group and IBD group in epithelial cells (FDR < 0.05). (Control *n* = 13, IBD *n* = 13). (H) Volcano map shows the number of DEGs in macrophage between control group and IBD group (|log_2_FC| > 0.25 and FDR < 0.05). (Control *n* = 13, IBD n = 13). (I) KEGG enrichment analysis of DEGs between Control group and IBD group in macrophage (FDR < 0.05). (Control *n* = 13, IBD *n* = 13). (J) Volcano map of DEGs between the Control group and IBD group (|log_2_FC| > 0.25 and FDR < 0.05). (Control *n* = 3, IBD *n* = 3). (K) GO‐enriched chord diagram of DEGs between the IBD group and OPR group (FDR < 0.05). (IBD *n* = 3, OPR *n* = 3). Heatmap analysis of the expression and regulation of actin cytoskeleton (L), gut microbiome (M), intestinal barrier function (N), glycolysis‐related pathway (O), pyroptosis (P), and STING‐related pathway (Q) in Control, IBD, and OPR groups. (Control *n* = 3, IBD *n* = 3, OPR *n* = 3). t‐SNE, t‐distributed stochastic neighbor embedding.

Afterwards, we subjected colon tissues from mice in the healthy control, IBD model, and OPR treatment groups to RNA‐seq analysis. As shown in Figure S32A, the correlation heatmap indicated that high intra‐group data correlation and significant inter‐group variability in the healthy control, IBD model, and OPR groups. The number of DEGs between the control group and the IBD group was 4464, and the number of DEGs between the IBD group and the OPR group was 4211 (*p* < 0.05 and |log_2_FC| > 1) (Figure S32B). The number of DEGs between the Control and OPR group was 341, which indicated that the overall gene expression pattern of OPR‐treated mice was close to that of healthy mice. The DEGs between the control and IBD groups were further analyzed using volcano plots, which showed that 3460 DEGs were upregulated and 1004 DEGs were downregulated in the IBD group compared to the control group (Figure 7J). To further elucidate the mechanism of OPR for IBD, we used the Gene Ontology (GO), KEGG to analyze the differentiation of genes between the OPR and IBD groups. As shown in Figure 7K, GO enrichment showed that the DEGs between OPR group and IBD group were mainly enriched in oxidative stress, bacterial infectious disease, adherens junction, cAMP signaling pathway, TNF signaling pathway, pyroptosis, and other signal pathways. KEGG enrichment showed that the DEGs between OPR group and IBD group were mainly enriched in response to bacteria infectious disease, glycolysis, STING‐related pathway, apoptosis, NF‐κB signaling pathway, TNF signaling pathway, PI3K‐Akt signaling pathway, mTOR signaling pathway, and adherens junction (Figure S32C). Subsequently, heat map analysis was performed on pathway‐related genes such as regulation of actin cytoskeleton (Figure 7L), gut microbiome (Figure 7M), intestinal barrier function (Figure 7N), glycolysis‐related pathway (Figure 7O), pyroptosis (Figure 7P), and STING‐related pathway (Figure 7Q). The levels of *UCP2*, *GSDME*, and *STING* mRNA in mouse colon samples were consistent with the protein levels detected in the previous experiments, and OPR significantly down‐regulated the expression of *UCP2*, *GSDME*, and *STING* genes in the colon tissue of IBD mice (Figure S33). In summary, these lines of evidence suggest that mitochondrial dysfunction exists in IBD, leading to IEC pyroptosis and subsequent activation of the STING pathway in macrophages. OPR can significantly protect mitochondria to reduce pyroptosis of IECs, and inhibit macrophage activation. These single‐cell sequencing and transcriptome clues are consistent with our experimental evidence, fully verifying that OPR achieves efficient treatment of IBD by repairing mitochondria of IECs and eliminating ROS. Moreover, OPR can also promote metabolic reprogramming (through glycolysis regulation) of IECs and achieve resistance to toxins of *Clostridium difficile*.

## DISCUSSION AND CONCLUSION

CDI usually occurs along with intestinal flora imbalance after patients receive antibiotic treatment, and has been recognized as a global clinical problem. Patients with IBD are at significantly increased risk of CDI. CDI greatly exacerbates IBD symptoms, leading to a significant increase in treatment failure and mortality in IBD. The mortality rate directly caused by CDI is approximately 5%, and the mortality rate of IBD complicated CDI is 15% to 25% [39]. To combat CDI, a large number of treatment strategies have been explored, mainly involving antibiotics, fecal microbiota transplantation, and toxin‐targeted therapy. In clinical practice, the first‐line treatment for CDI is vancomycin and fidaxomicin, of which vancomycin is more widely used due to its low cost. Vancomycin is used as a “last line drug,” generally to treat serious infections that have failed to respond to all other antibiotics. However, vancomycin not only has serious side effects, but also, as a general antibiotic, causes serious dysbacteriosis, ultimately leading to the recurrence of CDI. After the first attack of CDI, the second recurrence rate (8 weeks post the first CDI attack) is 15%–30%, and the third (8 weeks post the second CDI attack) is 45%–65%. The recurrence rate increases with each attack, forming a vicious cycle of CDI recurrence [40]. Fecal microbial transplantation involves transplanting fecal material from healthy individuals into the gastrointestinal tract of patients, which introduces probiotic flora to inhibit *Clostridium difficile* and restore intestinal microbial diversity. Although fecal microbial transplantation has shown high efficacy in the treatment of CDI, the potential risk of infectious transmission greatly limits the application of this method. Targeted therapy against *Clostridium difficile* toxins is an effective treatment strategy, such as the TcdB monoclonal antibody‐Bezlotoxumab and the emerging DLD‐4, but further research is needed to optimize its efficacy and safety [41]. In general, these methods for treating CDI are not compatible with the treatment of IBD, and may even produce a mutually deteriorating cycle with the treatment of IBD, leading to a dilemma in the prognosis of CDI and IBD comorbidities. Taking antibiotics complicated by first‐line IBD treatment (anti‐inflammatory drugs) as an example, treatment conflicts and toxic effects are inevitable during the treatment of IBD‐CDI. Antibiotic treatment of CDI further destroys the intestinal flora and aggravates the IBD condition, while immunosuppressant treatment of IBD induces systemic immunosuppression and aggravates CDI. This bidirectional pathological interference may lead to alternating aggravation of symptoms. Therefore, a treatment strategy that is compatible with the treatment of IBD and the elimination of the *Clostridium difficile* toxicity is essential for the treatment of IBD and CDI complicated IBD by precisely regulating the intestinal microecology and immune balance.

UCP2 has been known as a critical regulator for many mitochondria dysfunctional diseases. In type 2 diabetes (T2DM), GP treats T2DM by inhibiting UCP2 to restore MMP and mitochondrial function [18]. In CRC, UCP2 overexpression drives the Warburg effect and promotes cancer cell proliferation [42]. During aging, UCP2 defects inhibit glycolysis and enhance the ability of aging cells to resist oxidative stress, delaying aging [43]. However, the role of UCP2 in IBD remains obscure. In this study, we identify UCP2 as a critical metabolic regulator in IBD pathology for the first time. The positive correlation between UCP2 expression and disease severity in both human IBD intestinal tissue and mouse models underscores its pivotal role. UCP2 disrupts mitochondrial function by causing the collapse of MMP and reducing ATP synthesis, while simultaneously reprogramming cellular metabolism towards glycolysis. This metabolic shift results in L‐LA accumulation and cytoplasmic acidification, rendering IECs more vulnerable to TcdB. By targeting UCP2, our study addresses the core mechanisms driving IBD progression and its complications, offering a more precise therapeutic approach compared to conventional regimens that are trapped in the “IBD‐CDI” vicious cycle. OPR can maintain stability in the stomach and selectively release the UCP2 inhibitor‐GP in the IBD lesions, which enhances treatment specificity and reduces potential off‐target effects. Moreover, the mitochondrial targeting of OPR further ensures that the therapeutic action is precisely localized to mitochondria, optimizing the restoration of mitochondrial function in IECs. In IBD mouse models, OPR demonstrated remarkable therapeutic efficacy. By alleviating pyroptosis in IECs and inhibiting the activation of the macrophage STING pathway, OPR addresses the underlying cellular and immune dysregulation in IBD. The reduction in L‐LA levels and restoration of intracellular pH not only improve the metabolic state of IECs but also enhance their resistance to TcdB, effectively tackling the IBD‐CDI co‐infection problem. The superiority of OPR over first‐line clinical therapeutic drugs highlights its potential to revolutionize IBD treatment. In conclusion, this study breaks new ground in IBD treatment by targeting UCP2 and developing the innovative OPR system. The findings provide a novel treatment paradigm for IBD and IBD‐CDI syndrome.

## METHODS

### Human samples

All patients participating (the samples of patients) in this study were from Xiangya Hospital, Central South University. Lesioned colon tissues from three IBD patients and non‐inflamed colonic tissue from three CRC patients (used as healthy controls) were used for experiments. All the samples of colon tissues were collected immediately as the colon was resected in surgery. All procedures performed in the study involving human participants (the samples of patients) were in accordance with the ethical standards of the Ethics Committee of the Xiangya Hospital.

In this study, due to significant ethical and practical challenges in obtaining healthy adult colonic mucosal tissue samples (which would require invasive colonoscopic biopsies in healthy volunteers), we utilized histologically normal adjacent tissue from CRC resection specimens as “healthy” controls for IBD. These adjacent tissues were harvested from macroscopically normal mucosa at least 5 cm from the tumor margin and were confirmed by pathological examination to be free of malignant cells or significant inflammation. We fully acknowledge the potential limitations associated with such control samples; however, rigorously screened adjacent tissue from cancer resections remains one of the most feasible sources of control samples for IBD mechanistic studies under current ethical and technical constraints. All conclusions of this study are interpreted cautiously within this methodological context.

### Cells and reagents

HT29 cell line (RRID: CVCL_A8EZ) and RAW264.7 cell line (RRID: CVCL_0493) were purchased from the National Collection of Authenticated Cell Cultures of China. The cell lines were authenticated for the described experiments and have not been previously reported as misidentified or contaminated. The cell lines were free of mycoplasma contamination (confirmed by the supplier). The reagents used in this study are detailed in Table S1. The antibodies used in this study are detailed in Table S2 (with RRID).

### Synthesis and characterization of OPR

0.375 g of glycine was dissolved in 70 mL of PBS, 0.675 g of GP was dissolved in 30 mL of anhydrous ethanol, then the two solutions were mixed in a flask by stirring mixing. The final concentration of glycine and GP was 50 mM and 30 mM, respectively. The pH of the mixed solution was adjusted to 2.0 to start the Schiff base reaction. Then the reaction was carried out in a 50°C water bath for 24 h. After reaction, the solution was transferred into centrifuge tubes, and the supernatant was discarded after centrifugation (12,000 × *g*, 10 min). The precipitate was washed with ddH_2_O for five times to remove the unreacted impurities. After washing, the solution was centrifuged, and the supernatant was discarded after centrifugation (12,000 × *g*, 10 min). The precipitate was dialyzed against ddH_2_O for 24 h; the ddH_2_O was refreshed every 4 h. For long term storage, the dialyzed solution was centrifuged (12000 × *g*, 10 min) and the precipitate was freeze‐dried (−20°C, vacuum, 24 h). The lyophilized powder was stored at −20°C. For concentration determination, 1 mL dialyzed solution was collected, centrifuged, and dried to get the weight of the drug in the 1 mL solution. Prior to sample submission and formal experiments, a 30‐min sonication is conducted to ensure the formation of a solution system with uniformly dispersed particles.

### Synthesis and characterization of GPA

0.87 g of arginine was dissolved in 70 mL of PBS, 0.675 g of GP was dissolved in 30 mL of anhydrous ethanol, then the two solutions were mixed in a flask by stirring mixing. The final concentration of arginine and GP was 50 mM and 30 mM, respectively. The following process is the same as the OPR synthesis.

### Synthesis and characterization of OPR‐FITC

Weighed 10 mg of the above synthesized OPR and 12.5 mg of polyethylene glycol were added to 10 mL of Tris (hydroxymethyl) aminomethane (pH = 8.5) buffer solution, mixed well, and reacted at room temperature for 12 h. Centrifuged and discarded the supernatant, ddH_2_O was washed three times. The precipitate was dissolved with 8 mL of ddH_2_O, and 2 mg of fluorescein isothiocyanate (FITC) was dissolved in 2 mL of DMSO. When it was dissolved, it was mixed with the dissolved precipitate, and then the reaction was carried out for 5 h at room temperature, wrapped in tin foil to avoid light. After the reaction, centrifuge and discard the supernatant, and the precipitate was fixed to 10 mL with ddH2O, and after ultrasonication and mixing, the solution was dialyzed for 24 h (with 4–6 water changes) to obtain OPR‐FITC. The GP‐FITC and GPA‐FITC were synthesized with the same protocol.

### Scavenging of ROS

The ability of OPR to scavenge O_2_·^−^ was examined by the nitrogen blue tetrazolium (NBT) method. In the presence of methionine, riboflavin was reduced to O_2_·^−^. O_2_·^−^ bound to NBT to form a blue colored methionine with maximum absorption at 560 nm. Different concentrations of OPR (1, 2, 4, 8, 16 μg/mL) were mixed with methionine (100 μM), riboflavin (20 μM), NBT (10 mM), PBS (0.01 M, pH 7.4), and deionized water. Then, it was exposed to UV light for 7 min. Finally, O_2_·^−^ clearance was measured by inhibiting the photochemical reduction of NBT. The H_2_O_2_‐scavenging activity of OPR was measured by UV/Vis spectrophotometry. Three milliliters of OPR (10 μg/mL) was mixed with 1 mL of H_2_O_2_ at concentrations of 150, 300, 600, 1200, 2400, and 4800 mM, and then incubated in the dark for 12 h. H_2_O_2_ scavenging was determined by examining the characteristic UV absorption curve of OPR. The ·OH scavenging activity of the OPR was determined using the disodium terephthalate (TA) method. TA is able to capture the ·OH generated by the reaction between H_2_O_2_ and ferrous sulfate, which reacts to produce 2‐hydroxyterephthalic acid, which is fluorescent (Ex:320 nm, Em:425 nm). The OPR reduces the conversion of TA to 2‐hydroxyterephthalic acid by scavenging ·OH, decreasing the fluorescence intensity.

### Characterization of OPR

Simulated gastric fluid (SGF): Weigh 1 g pepsin and dissolve it in 90 mL of ddH_2_O, then adjust the pH to 1.5 with hydrochloric acid and set the volume to 100 mL. SIF: Weigh 1.36 g of monobasic potassium phosphate and dissolve it with 100 mL of ddH_2_O. Another 2 g of trypsin is dissolved in an appropriate amount of ddH_2_O. After mixing the two, NaOH was used to adjust the pH to 6.5, and then the volume was adjusted to 200 mL. The OPR is incubated with SGF for 4 h to simulate gastric emptying time to obtain SGF‐OPR. SGF‐OPR is used for XPS assay and TEM observation for the determination of OPR and SGF‐OPR, and the determination of OPR free radical scavenging capacity in vitro after SGF treatment. TECNAI G2 high‐resolution TEM was utilized to capture TEM images. XPS measurements (VG ESCALAB MKII) were used to analyze the elemental states of OPR.

### OPR degradation to GP

OPR was incubated in SIF at 37°C for 0, 2, 4, 6, 8, 10, 12, 16, 20, 24, 36, 48, 60, 72, 96, 120, 144 and 168 h. OPR was incubated in SGF at 37°C for 0, 2, 4 h. The content of GP at each time point was determined by Agilent/1290 Infinity Ⅱ analytical liquid chromatography purification system. For color change observation, 3 mL solutions at each time point were collected into transparent glass bottles for photography. The percentage of OPR release at each time point was recorded and used for nonlinear fit to calculate the half‐life of OPR.

### Construction of DSS‐Induced IBD mouse model and related treatments

Eight‐week‐old male C57BL/6J (23–25 g) mice were housed in cages of 4 mice each and acclimatized for 1 week prior to inclusion in the study. Mice were induced with 3% DSS for 7 days to construct an IBD model and then given normal water. The control group was provided with normal water only to healthy mice. For other treatment groups, on Days 7, 9, and 11, mice were orally administered PBS or OPR (5 mg/kg), GP (5 mg/kg), 5‐ASA (30 mg/kg), DEX (1 mg/kg), and UPA (10 mg/kg). INF (5 mg/kg) was injected intravenously. Finally, on Day 13, the animals were euthanized. The intestinal tissues of the mice were removed for the next step of the assay.

In the experiment to verify the organ‐targeted distribution of OPR, healthy and IBD mice were used for oral or intravenous injection of OPR‐FITC. Twelve hours later, the tissues of heart, liver, spleen, lung, kidney, and intestine of mice were taken for stereofluorescence microscopy (M205FCA) purchased from Leica (Germany). In the experiment to verify the oral colon targeting duration of OPR, healthy or IBD mice were given OPR‐FITC orally, and the colon fluorescence intensity of mice was detected at 1, 3, 6, 9, 12, 24, 48, and 72 h, respectively. The animal experiment process involved in this study was approved by the Ethics Committee of Experimental Animal Welfare of Central South University.

### Construction of IBD complicated CDI mouse model and related treatments

Eight‐week‐old male C57BL/6J (23–25 g) mice were housed in cages of 4 mice each and acclimatized for 1 week prior to inclusion in the study. Mice were induced with 3% DSS for 7 days to construct an IBD model, and then 10^6^ CFU were given orally by gavage on day 7 to construct an IBD complicated CDI model. The control group was provided with normal water only to healthy mice. For the model and OPR groups, mice were orally administered PBS or OPR (5 mg/kg) on Days 7, 9, and 11. For the F‐I group, Fidaxomicin (30 mg/kg) was given orally for three consecutive days on Days 7–9, and INF (5 mg/kg) was given by tail vein injection for three consecutive days on Days 10–12.

### *In vitro* adhesion properties of OPR

*In vitro* adhesion characterization of OPR‐FITC for in vitro imaging adhesion experiments. Polystyrene plates were coated with MUC2 isolated from porcine stomach or human TF to simulate healthy and inflamed colonic mucosa, respectively. One mL of MUC2 at a concentration of 30 mg/mL was dissolved in HBSS. The mixture was then warmed at 25°C for 2 h with polystyrene plates and gently shaken. It was dissolved in PBS containing 500 μg/mL TF and then incubated with polystyrene plates at 37°C overnight. Polystyrene plates incubated with PBS overnight at 37°C were used as controls. Next, the polystyrene plates were rinsed with PBS. OPR‐FITC was dispersed in an aqueous solution at a concentration of 80 μg/mL and added to the polystyrene plates. Gently shake and incubate for 1 h at room temperature. After washing with PBS, the chamber slides were imaged using a fluorescence microscope. The adhesion assay of GPA‐FITC was performed with the same protocol.

### Determination of OPR and GP in mouse plasma by HPLC

OPR and GP were dissolved in water to a concentration of 5 mg/kg, and mice were injected orally or by tail vein, and blood samples were collected before and 5, 10, 30, 60, 120, 180, 240, 360, 540, 720, 1440, 2880, and 4320 min after the administration of OPR and GP, and plasma was obtained by centrifuging the supernatant at 12,000 × *g* for 10 min to obtain a plasma Sample. The plasma was mixed 2:1 with sulfate esterase and incubated at 37°C for 30 min, and then the GP content at each time point was determined by Agilent/1290 Infinity II Analytical Liquid Chromatography Purification System. Calibrators were prepared by adding different concentrations of GP standards to blank plasma, followed by the addition of pH 5.0 acetate buffer. The rest of the procedure was the same as described above for plasma samples. The standard concentrations were 0.1, 0.2, 0.3, 0.6, 1.3, 2.5, 5.0, and 10 μg/mL GP. The regression equation was obtained by plotting the standard curve of GP, and the concentration of GP was obtained as the ratio of peak area.

### Assessment of DAI

The DAI was utilized to assess the effectiveness of OPR in the IBD model. DAI measures the percentage of weight loss. Score the results of stool consistency and stool bleeding, and calculate the final DAI by dividing the total score by 3 (Table S4).

### Histological analysis

To assess the severity of IBD and other relevant indicators in mice, intestinal tissue samples were obtained from each group of mice and subjected to H&E staining and IF assay. Antibodies used for IF are detailed in Table S2. TUNEL assay was performed using the Apoptosis Detection Kit. ROS staining of tissue frozen sections was performed using DHE.

Tissue paraffin sections were prepared and then baked at 65°C for 30 min, followed by deparaffinization using xylene and rehydration using gradient ethanol. After treatment, the sections were permeabilized with 0.5% TritonX‐100 for 15 min at room temperature. Thermal repair was then performed using EDTA antigen repair solution. 10% normal goat serum was added for 60 min at room temperature for containment. Diluted primary antibody was then added and incubated overnight at 4°C in the refrigerator. The next day, fluorescent secondary antibody was added and incubated for 1 h at room temperature. The nuclei were restained by adding DAPI, and the slides were sealed with fixation medium containing an anti‐fluorescence quencher. Finally, images were observed and captured using a fluorescence microscope. Specific conditions were adjusted according to the manufacturer's instructions.

### Bacterial culture

*C. difficile* VPI10463 (ATCC43255) was purchased from the American Typical Cultures Conservation Center (ATCC), and cultures were prepared using Brain Heart Infusion Broth supplemented with 5 g/L yeast extract, 0.1% L‐galactose and 0.1% sodium taurate.

*Bacteroides fragilis* 2‐078382‐3 (ATCC43858) was purchased from the American Typical Cultures Conservation Center (ATCC); cultures were prepared using Brain Heart Infusion Broth supplemented with 5 g/L yeast extract, 0.5 g/L cysteine, 5 mg/L heme, and 1 mg/L vitamin K. The culture was prepared using Brain Heart Infusion Broth supplemented with 5 g/L yeast extract, 0.5% L‐galactose, and 0.1% sodium taurate.

### Quantification of the C. difficile burden

To quantify *C. difficile* burden, colonic contents were suspended in ethanol for serial dilutions and incubated anoxically at 37°C in the culture solution described above (the culture solution was supplemented with 16 mg/L cefoxitin and 500 mg/L D‐cycloserine). After 48 h of incubation, colony‐forming units were counted and standardized to fecal weight.

### Purification of BFT

After removal of bacteria by centrifugation at 5000×*g* for 40 min at 4°C, the supernatant was precipitated with 70% saturated ammonium sulfate for 2 h and then incubated overnight at 4°C. The precipitate was collected by centrifugation at 7000 × *g* for 30 min and then dissolved in 100 mL of Tris‐HCl buffer with 1 mg/mL TPCK and dialyzed with buffer A (0.05 M Tris‐HCl) overnight at 4°C. This dialysate was loaded onto a QSepharose anion exchange column. The column was washed with buffer A, and the bound material was eluted by applying a 0–0.5 M NaCl gradient. A 10 mL gradient was collected, and the toxin‐containing gradients were concentrated using a 10‐kDa centrifugal filter, and the buffer was exchanged to buffer B (0.05 M Tris‐HCl, 1.5 M NaCl). The concentrated samples were then added to a phenyl agarose chromatography column equilibrated in buffer B. The column was washed sequentially with 1.5, 1.0, and 0.5 M NaCl in buffer A and finally eluted with 25% ethanol. The column eluate monitored by UV absorption at 280 nm was subjected to graded separation, and the toxin‐containing fractions were combined. After dialyzing 1 L of buffer A overnight at 4°C, the BFT fraction was applied to a MonoQHR5/5 anion‐exchange column equilibrated in buffer A. The column was washed with the same buffer. After washing with the same buffer, the bound material was eluted with a 0–0.5 M NaCl gradient. The BFT activity of the column eluate was monitored by the HT29 cell assay.

### Cell culture

HT29 was cultured using RPMI‐1640 medium containing 10% fetal bovine serum, and RAW264.7 macrophages were cultured in DMEM containing 10% fetal bovine serum. All cells were cultured at 37°C in 5% CO_2_. Cells were purchased from the Typical Culture Preservation Center of China.

HT29 cells were inoculated into 6‐well plates at 10^6^ per well and then incubated for 24 h. Subsequently, 2 mM H_2_O_2_ was added to construct an oxidative stress cell model, and then OPR or GP at a concentration of 10 μg/mL was dispersed in the medium and incubated at 37°C for 24 h.

HT29 cells were inoculated into 6‐well plates at 10^6^ per well and then incubated for 24 h. Subsequently, 30 ng/mL TcdB was added to construct a cell model of *C. difficile* infection, and then OPR or GP at a concentration of 20 μg/mL was dispersed in the medium and incubated at 37°C for 24 h.

HT29 cells are seeded into 6‐well plates at 10^6^ per well and then incubated for 24 h. Subsequently, 2 mM H_2_O_2_ was added, followed by 30 ng/mL TcdB for 2 h to construct an oxidative stress and CDI combined cell model, and then OPR or GP at a concentration of 20 μg/mL was dispersed in the culture medium and incubated at 37°C for 24 h.

A Transwell system was constructed in which HT29 cells were inoculated into the upper chamber at 10^6^ per well and RAW264.7 cells were inoculated into the lower chamber at 10^6^ per well, and the oxidative stress model was induced in the upper chamber using 2 mM H_2_O_2_, and then OPR or GP at a concentration of 10 μg/mL was added to the upper chamber for a total of 48 h. RAW264.7 cells in the lower chamber were collected for subsequent assays.

### Construction of UCP2‐OE plasmid and transfection into HT29 cells

PCR amplification of the CDS sequence of UCP2 (NM_003355.3) was performed using primers with NheI/XhoI enzyme cleavage sites. The CDS sequence of UCP2 was inserted into the NheI/XhoI restriction enzyme‐digested pcDNA3.1(+) vector using T4 DNA ligase. The construction of pcDNA3.1‐UCP2 (UCP2‐OE) plasmid was verified with agarose gel electrophoresis (Table S5).

The UCP2‐OE plasmid was transfected into HT29 cells with Lipofectamine 3000 kit following the manufacturer's instructions. To evaluate the transfection efficiency, the plasmid was labeled with Cy3‐dUTP and the fluorescence in cells was observed 8 h after plasmid transfection. The cells were collected 24 h after transfection to test the *UCP2* mRNA levels with qPCR. The cells were collected 48 h after transfection to test the UCP2 protein levels with WB. The cells with confirmed UCP2 overexpression were used for further experiments.

### ROS scavenging in cells

To measure ROS levels in live cells, a DCFH‐DA probe was added to each well using the recommended concentration according to the instructions and incubated for 25 min. Cells were washed three times with PBS, then Hoechst was added to re‐stain the nuclei, and images were taken using a fluorescence microscope. For quantitative analysis, cells were first collected, washed three times in PBS buffer, and then resuspended in DCFH‐DA probe working solution for 25 min. After three washes in cold PBS, cells were analyzed by flow cytometry.

### Mitochondrial ROS scavenging in cells

To measure mitochondrial ROS levels in live cells, we used the MitoSOX probe added to each well at the recommended concentration according to the instructions and incubated for 10 min, washed the cells three times with HBSS, and added Hoechst to re‐stain the nuclei, and then captured the images using a fluorescence microscope. For quantitative analysis, cells were first collected, washed three times in PBS buffer, and then resuspended in MitoSOX probe working solution for 15 min. After three washes in HBSS, cells were analyzed by flow cytometry.

### MMP detection in cells

To measure the level of MMP in living cells, we used the JC‐1 probe added to each well at the recommended concentration according to the instructions and incubated for 20 min, then washed the cells three times with JC‐1 staining buffer, restained the nuclei using Hoechst, and performed fluorescence microscopy to take images. For quantitative analysis, cells were first collected, washed three times in PBS buffer, and then resuspended in JC‐1 probe working solution for 20 min. After three washes in JC‐1 staining buffer, cells were analyzed by flow cytometry.

### ATP level measurement in cells

To measure the ATP level in living cells, the cells were first washed three times with PBS, then lysed by adding lysis buffer from the Enhanced ATP Assay Kit; after sufficient lysis, centrifugation was performed to take the supernatant and the ATP assay reagent for rapid mixing and reading the signal using a luminometer. The ATP concentration was calculated from the luminescence signal.

### OCR and ECAR assays

HT29 cells were seeded into a 96‐well clear bottom black polystyrene microplate and incubated with different treatments for 24 h. The OCR of the cells was determined by a fluorometric OCR assay kit following the manufacturer's instructions. Briefly, 100 μL OCR‐specific probe was added to each well and the microplate was read on a fluorescence microplate reader every 10 min. At 20, 50, 80 min, 10 μL of Oligomycin A, FCCP, and Rotenone were added to each well, respectively. The ECAR of the cells was determined by a fluorometric ECAR assay kit following the manufacturer's instructions. Briefly, 100 μL ECAR‐specific probe was added to each well and the microplate was read on a fluorescence microplate reader every 10 min. At 20, 50, 80 min, 10 μL of glucose, Oligomycin A, and 2‐DG were added to each well, respectively. The assays were completed at 110 min.

### Co‐localization of drugs and mitochondria in cells

We first used 10 μg/mL OPR‐FITC to co‐incubate with HT29 cells for 24 h. Then they were incubated with MitoTracker working solution (200 nM) for 30 min. After replacing the staining solution using fresh pegylated medium, images were taken under a fluorescence microscope.

### Apoptosis analysis in cells

To detect apoptosis using AnnexinV‐FITC/PI, cells were collected and washed three times with PBS, then stained with AnnexinV/PI working solution for 20 min. Cells were analyzed using a Novocyte 3130 flow cytometer (Agilent).

### Live/dead staining in cells

Calcein‐AM and PI were used to perform live‐dead cell staining by first washing the cells three times with staining buffer, then staining them with Calcein‐AM/PI working solution for 20 min, and finally washing the cells three times with staining buffer and taking images using a fluorescence microscope.

### Immunofluorescence staining in cells

Cells from different treatments were washed three times using PBS and then fixed using 4% paraformaldehyde for 30 min at room temperature. They were then permeabilized using 0.3% TritonX‐100 for 15 min. 10% normal goat serum was added for 60 min at room temperature for containment. Diluted primary antibody was then added and incubated overnight at 4°C in the refrigerator. The next day, fluorescent secondary antibody was added and incubated for 1 h at room temperature. The nuclei were restained by adding DAPI, and the slides were sealed with fixation medium containing an anti‐fluorescence quencher. Finally, images were observed and captured using a Zeiss Elyra7 Lattice Light Microscope. Specific conditions were adjusted according to the manufacturer's instructions.

### Safe concentration screening and antitoxin effect assay (CCK‐8 assay)

In the safe concentration screening assay, HT29 cells were incubated in 96‐well plates and treated with 0.3–300 μg/mL OPR for 24 or 48 h. Then 10 μL of CCK‐8 reagent was added to each well to incubate with the cells for 2 h. Absorbance values at 450 nm were detected by using a multimode microplate reader.

In the assay of the antitoxin effect of OPR, HT29 cells were incubated in 96‐well plates and treated with 2 mM H_2_O_2_ + 30 ng/mL TcdB, 2 mM H_2_O_2_ + 7 ng/mL Stx2, 2 mM H_2_O_2_ + 500 ng/mL SAH, 2 mM H_2_O_2_ + 5 ng/mL BFT, and then 0.5–80 μg/mL of OPR or GP were added for a total of 24 h of incubation. Finally, 10 μL CCK‐8 reagent was added to each well. After a 2‐h incubation, the absorbance value of each well at 450 nm was detected using a multimode microplate reader. Unlike the other three toxins, BFT exhibits a proliferative‐like cytotoxic effect due to its ability to aberrantly activate pro‐proliferative genes.

### CellTiter‐Glo® luminescent cell viability assay

HT29 cells were seeded into 96‐well plates and then incubated for 24 h. Cells were treated with 2 mM H_2_O_2_, 30 ng/mL TcdB, and 0.5–80 μg/mL OPR or GP for 24 h. Cells were incubated for 10 min at room temperature after adding 100 μL of CellTiter‐Glo® Reagent to each well. Subsequently, the signal was read using a luminometer, and the dose–response curves of OPR and GP were plotted.

### LysoTracker staining in cells

LysoTracker was used to perform staining of acidic endosomes. Cells were first washed three times with PBS, then stained with LysoTracker working solution for 40 min, and at the end of the staining, cells were washed three times with PBS, then Hoechst was added to restain the nuclei, and images were taken using a fluorescence microscope.

### Intracellular pH analysis

Intracellular pH measurements were performed using CarboxySNARF‐1AM, a cell membrane‐permeable pH probe that exhibits a distinct pH‐dependent migration of its emitted light from yellow–orange to deep‐red fluorescence under acidic and alkaline conditions, respectively. This pH dependence allows us to quantify pH using the ratio of fluorescence intensity at the two emission wavelengths of the dye (580 and 640 nm). Briefly, cells were washed three times using PBS, followed by staining the cells with 5 μM CarboxySNARF‐1AM for 30 min. The cells were then washed three times using PBS and assayed using a luciferase marker.

### Cytoskeleton staining

Staining of the cytoskeleton using FITC‐labeled ghost pen cyclic peptide. Phalloidin, a cyclic heptapeptide toxin derived from the muscaria Amanitaphalloides, selectively binds to filamentous actin F‐actin with high affinity, thus allowing staining of the cytoskeleton. Briefly, we performed staining of cell and tissue paraffin sections for 40 min using 100 and 200 nM ghost pen cyclic peptides, respectively. The rest was performed according to the instructions.

### WB analysis

We used RIPA to lysed protein components in tissues or cells and determined the protein content by BCA assay. Protein samples were denatured at a temperature of 95°C for 7 min. electrophoresis on SDS‐PAGE gels and then transferred to PVDF membranes. Primary antibody was incubated overnight at 4°C. The following day, the secondary antibody was added and incubated for 1 h at room temperature. The membranes were finally visualized in ECL luminescent solution, and images were obtained on the Bio‐Rad gel imaging system (Bio‐Rad). The primary antibodies involved are detailed in Table S2.

### Enzyme‐linked immunosorbent assay (ELISA) analysis

The levels of TNF‐α, IL‐6, and IL‐β in the intestinal tissues of mice from different treatment groups were measured using specific ELISA kits. Mouse intestinal tissue homogenates were prepared at 4°C. Each sample was centrifuged at 10,000 × *g* for 10 min at 4°C. Levels of each indicator in the supernatant were measured using ELISA. Specific steps for implementation can be found in the manufacturer's instructions.

### Biochemical assays

Colon tissue homogenates were used to detect levels of NAD^+^/NADH, ADP/ATP, Hexokinase 2, PFK activity, MPO, MDA, and L‐LA. And cell lysate was used to detect levels of L‐LA. The assays were performed according to the manufacturer's instructions.

### Quantitative PCR analysis

Genomic DNA was extracted from each group of mouse feces using the Fecal Genomic DNA Extraction Kit. Total RNA from mouse colon tissue was extracted using RNAiso Plus, and RNA was dissolved in RNase‐free water. The genomic DNA from the sample was then removed using gDNA Eraser reagent. The reverse transcription reaction was performed with a 20 μL system as follows: Template RNA 10 μL (100 ng/μL); PrimeScript RT Enzyme Mix I 1 μL; RT Primer Mix 1 μL; 5 × PrimeScript Buffer 4 μL; H_2_O 4 μL. After testing for concentration and integrity, TB Green Premix Ex TaqTM was used for quantitative PCR. The reaction was performed using a 20 μL system as follows: 10 μL of 2× mixture; 0.4 μL of each of the primers F/R (10 μM); 0.4 μL of ROX (50×); Template DNA (60 ng/μL) 1 μL; H_2_O 7.8 μL. Applied Biosystems Step One Plus instrument. A two‐step amplification program was used, starting with a 95°C pre‐denaturation for 30 s, followed by 40 cycles of 95°Cdenaturation for 5 s and 60°C annealing and extension for 30 s steps. Expression levels were determined using the 2^−ΔΔCT^ method with 16S as the internal reference gene. The primer sequences are detailed in Table S3.

### Single‐cell sequencing dataset analysis

Raw sequencing data (fastq files) and sample metadata for dataset GSE266616 were downloaded from the Gene Expression Omnibus (GEO) database. Cell Ranger (v7.0) was employed for sequence alignment, cell barcode, and UMI counting, generating a gene‐cell expression matrix. To mitigate the impact of batch effects and donor‐specific variability on clustering analysis, we systematically performed data integration and batch correction after generating the gene‐cell expression matrix. Specifically, we applied the integration pipeline in Harmony to harmonize samples from different patients and inflammatory sites. Following integration, we used community‐based clustering and marker‐gene analysis to identify five major clusters: IECs (marked by EPCAM), B cells (marked by CD79), T cells (marked by CD3D), myeloid cells (marked by TYROBP), and stromal cells (marked by IGFBP7). Each major lineage was further categorized into multiple cell types. Cell annotation and further DEG analysis were performed using SingleR (v2.0) and Seurat (v5.0). GO and KEGG enrichment analysis of DEGs was performed using ClusterProfiler (v4.0).

### Transcriptomics analysis

Total RNA from mouse colon tissue was extracted using the TRIzol method, and RNA samples were analyzed for integrity and purity after extraction. Libraries were built using Illumina's NEBNext® UltraTM RNA Library Prep Kit. After passing the library test, different libraries were pooled according to the effective concentration and the target downstream data volume, and then sequenced by Illumina, and 150 bp paired‐end reads were generated. The index of the reference genome was constructed using HISAT2 v2.0.5, and the paired‐end clean reads were aligned to the reference genome using HISAT2 v2.0.5. Stringtie was used to calculate the number of reads mapped to each gene. Gene‐level read counts were obtained from the alignment results and used as input for differential expression analysis. Differential expression analysis between the two comparison combinations (three biological replicates per group) was performed using DESeq2 software (1.16.1). GO enrichment analysis of DEGs was achieved by clusterProfiler (3.4.4) software. The sequencing service and data analysis service were provided by Wekemo Tech Group Co. Ltd., Shenzhen, China.

### Gut microbiome analysis

The genomic DNA of the samples was extracted using CTAB, after which the purity and concentration of the DNA were detected using 1% agarose gel electrophoresis. An appropriate amount of the sample was taken in a centrifuge tube, and the sample was diluted to 1 ng/µL using sterile water. The samples were analyzed using the 515F (5′‐GTGCCAGCMGCCGCGGGTAA‐3′) and 806R (5′‐GGACTACHVGGGTWTCTAAT‐3′) primers for PCR amplification of the V4 variable region. After amplification and purification, the libraries were constructed using NEB Next® Ultra DNA Library Prep Kit library construction kit, and the constructed libraries were detected and quantified by Q‐PCR using Agilent 5400; after the libraries were qualified, they were up‐sequenced using the Illumina sequencing platform. The analysis process mainly involves the use of qiime tools import plug‐in to import the original sequence.fastq file into a file format that can be subsequently processed by QIIME2. Then the QIIME2 dada2 plug‐in was used for quality control, trimming, denoising, splicing, and chimera removal to obtain the final feature sequence table. Next, the QIIME2 feature‐classifier plugin was applied to compare the representative sequences of ASVs to the pre‐trained GREENGENES database of version 13_8 with 99% similarity (the database was pruned to the region of V4 based on the 515F/806R primer pair), and the taxonomic information table of the species was obtained. All contaminating mitochondrial and chloroplast sequences were subsequently eliminated using the QIIME2 feature‐table plugin. ANCOM, ANOVA, Kruskal–Wallis, LEfSe, and DESeq2 were employed to identify bacteria that differed in abundance between groupings and samples. Among them, Alpha diversity was measured by the Chao1 index. PCoA analysis was utilized to demonstrate Beta diversity and further compare the differences between sample groups. The sequencing service and data analysis service were provided by Wekemo Tech Group Co. Ltd., Shenzhen, China.

### Biocompatibility evaluation of OPR

To assess long‐term toxicity, healthy mice were orally administered OPR for 7 days or OPR for 24 h and analyzed for changes in organ histology by H&E staining. We used whole blood samples for hematological analysis. Parameters analyzed included blood and liver function (ALT, AST) and kidney function (BUN, CRE).

### Statistical analysis

All the data were presented as the mean ± SD. from a minimum of three independent experiments. Data analysis was performed using Image J (version 1.8.0), and GraphPad Prism Software Version 8.0 (GraphPad Prism). According to the normality results, an unpaired, two‐tailed Student's *t*‐test or Non‐parametric Mann–Whitney *U* test was used for two‐group comparisons. For comparisons of more than two groups, the data were analyzed using one‐way or two‐way analysis of variance (ANOVA) with Tukey's test and Sidak's multiple comparisons test. *p*‐value < 0.05 was considered to indicate a statistically significant difference. The *p* values and *n* values are given in the figures or corresponding legends.

For repeated measures data (weight change, DAI), the statistical significance was determined by two‐way repeated measures ANOVA (group × time), followed by Tukey's post hoc test for multiple comparisons. *p*‐value < 0.05 was considered to indicate a statistically significant difference.

For Kaplan–Meier survival curves, the data were analyzed using the log‐rank test (Mantel–Cox). *p*‐value < 0.05 was considered to indicate a statistically significant difference.

For Chao1 index (α diversity) analysis, the statistical significance was determined by the Kruskal–Wallis test followed by pairwise Dunn's test with Benjamini–Hochberg correction. FDR < 0.05 was considered to indicate a statistically significant difference.

For PCoA (β diversity) analysis, the statistical significance was determined by permutational multivariate ANOVA (PERMANOVA) with Benjamini–Hochberg correction. FDR < 0.05 was considered to indicate a statistically significant difference.

For the relative abundance of gut microbiota, the data were analyzed by Kruskal–Wallis test with Benjamini–Hochberg correction for multiple comparisons. FDR < 0.05 was considered to indicate a statistically significant difference.

For DEG analysis, the data were analyzed with DESeq2 package, *p* values were derived from the Wald test and were adjusted for multiple testing using the Benjamini–Hochberg correction. FDR < 0.05 was considered to indicate a statistically significant difference.

For GO/KEGG enrichment analysis, significantly enriched terms were identified based on a hypergeometric test, with *p* values adjusted for multiple comparisons using the Benjamini–Hochberg correction. Only terms meeting the threshold (FDR < 0.05) were displayed.

## AUTHOR CONTRIBUTIONS

**Wensheng Chen**: Investigation; writing—original draft. **Yuntao Zhang**: Investigation; writing—original draft. **Jue Wang**: Investigation. **Yiming Li**: Investigation. **Hao Wu**: Investigation. **Qiong Huang**: Investigation; conceptualization; writing—review and editing; project administration; supervision; funding acquisition. **Wei Wu**: Writing—review and editing; supervision. **Kelong Ai**: Conceptualization; project administration; supervision; resources; funding acquisition; writing—review and editing; writing—original draft. All authors have read the final manuscript and approved it for publication.

## CONFLICT OF INTEREST STATEMENT

The authors declare no conflicts of interest.

## ETHICS STATEMENT

The study was approved by the Ethics Committee of the Xiangya Hospital (Approval ID: 2024030265), and all participants provided written informed consent before taking part in the study.

## Supporting information


**Figure S1.** DEG analysis of the human source dataset [GEO]GSE179285.
**Figure S2.** Analysis of human colon tissues.
**Figure S3.** Synthesis and characterization of OPR and SGF‐OPR.
**Figure S4.** Color change of OPR under SIF and SGF conditions.
**Figure S5.** Half‐life calculation of OPR under SIF condition.
**Figure S6.** Targeting ability of OPR.
**Figure S7.** Distribution of GP‐FITC in IBD and healthy mice after oral administration.
**Figure S8.** Characterization of GPA.
**Figure S9.** Dosage screen of OPR based on the body weight change of mice after treatment.
**Figure S10.** Dosage screen of OPR based on the colon length change of mice after treatment.
**Figure S11.** Biocompatibility of OPR.
**Figure S12.** Inhibition effect of OPR and GP on TcdB.
**Figure S13.** Effect of OPR on cell energy metabolism.
**Figure S14.** Analysis of metabolic indicators in mouse colon.
**Figure S15.** Concentration screen of OPR based on the regulation effect of cell viability.
**Figure S16.** Concentration screen of OPR based on the effect of ROS clearance.
**Figure S17.** Effect of OPR on mitochondria protection.
**Figure S18.** Co‐localization analysis of OPR‐FITC and mitochondria.
**Figure S19.** Interaction analysis between OPR and mitochondrial outer membrane proteins.
**Figure S20.** Confirmation of UCP2‐OE transfection in HT29 cells.
**Figure S21.** Effect of OPR in UCP2‐OE transfected HT29 cells.
**Figure S22.** Effect on ROS clearance of different drugs in mouse colon.
**Figure S23.** Anti‐apoptosis effect of OPR.
**Figure S24.** Effect of OPR on pyroptosis pathway.
**Figure S25.** Effect of different drugs on UCP2 expression in mouse colon.
**Figure S26.** Effect of different drugs on BAX and C‐Cyt C expression in mouse colon.
**Figure S27.** Effect of different drugs on junction proteins in mouse colon.
**Figure S28.** Effect of different drugs on dsDNA release in mouse colon.
**Figure S29.** Effect of different drugs on inflammatory cytokine levels in mouse colon.
**Figure S30.** Effect of different drugs on MPO activity in mouse colon.
**Figure S31.** Analysis of human single‐cell sequencing database.
**Figure S32.** Analysis of mouse colon transcriptomic sequencing data.
**Figure S33.** Effect of OPR on the gene expression in mouse colon.

**Table S1.** List of reagents.
**Table S2.** List of antibodies.
**Table S3.** Primers used for quantitative real‐time PCR.
**Table S4.** Assessment of DAI.
**Table S5.** Verification of pcDNA3.1‐UCP2 plasmid construction.

## Data Availability

The 16S‐rRNA sequencing data have been deposited in NCBI under accession no. PRJNA1403170 (https://www.ncbi.nlm.nih.gov/sra/PRJNA1403170). The transcriptome sequencing data have been deposited in NCBI under accession no. PRJNA1402710 (https://www.ncbi.nlm.nih.gov/sra/PRJNA1402710). The data and scripts used are saved in GitHub (https://github.com/2819808502/OPR.git). Supplementary materials (figures, tables, graphical abstract, slides, videos, Chinese translated version, and update materials) can be found in the online DOI or iMeta Science http://www.imeta.science/.
