# Supplementary material for: Oral proton‐reprogrammed nanomedicine to break the inflammatory bowel disease‐Clostridium difficile infection vicious cycle
Source: Imeta. 2026 Feb 20;5(1):e70112. doi: 10.1002/imt2.70112 (PMC13726583; doi:10.1002/imt2.70112)
Supplement: Supplementary file 1 [file IMT2-5-e70112-s001.docx]

**Supporting information to**

**Oral proton-reprogrammed nanomedicine to break the inflammatory bowel disease-*Clostridium difficile* infection vicious cycle**

**Running title: UCP2 inhibition breaks the IBD-CDI vicious cycle**

Wensheng Chen^1,2#^, Yuntao Zhang^3,4#^, Jue Wang^3,4^, Yiming Li^2,3,5^, Hao Wu^2,3,5,7^, Qiong Huang^1,2*^, Wei Wu^2,5,6,8*^, Kelong Ai^3,4,9*^

^1^ Department of Pharmacy, Xiangya Hospital, Central South University, Changsha, 410008, China.

^2^ National Clinical Research Center for Geriatric Disorders, Xiangya Hospital, Central South University, Changsha, 410008, China.

^3^ Xiangya School of Pharmaceutical Sciences, Central South University, Changsha, 410078, China

^4^ Hunan Provincial Key Laboratory of Cardiovascular Research, Xiangya School of Pharmaceutical Sciences, Central South University, Changsha, 410078, China.

^5^ Department of Geriatric Surgery, Xiangya Hospital, Central South University, Changsha, 410008, China.

^6^ Department of General Surgery, Xiangya Hospital, Central South University, Changsha, 410008, China.

^7^ Department of Gastroenterology, Xiangya Hospital, Central South University, Changsha, 410008, China.

^8^ Key Laboratory of Biological Nanotechnology of National Health Commission, Changsha, 410008, China.

^9^ Key Laboratory of Aging-related Bone and Joint Diseases Prevention and Treatment, Ministry of Education, Xiangya Hospital, Central South University, Changsha, 410008, China.

^#^ These authors contributed equally: Wensheng Chen, Yuntao Zhang.

^*^ Correspondence: qionghuang@csu.edu.cn (Prof. Qiong Huang), [wuweixy@csu.edu.cn](mailto:wuweixy@csu.edu.cn) (Prof. Wei Wu), aikelong@csu.edu.cn (Prof. Kelong Ai)

**Figure S1.** DEG analysis of the human source dataset [GEO]GSE179285. (A) Volcano map shows the number of DEGs between Control group and IBD group (|log_2_FC| > 1 and FDR < 0.05). (Control n = 20, IBD n = 16). (B-D) GSEA between Control group and IBD group (Glycolysis (B), ROS-related pathway (C) and cytoskeletal protein regulation of actin cytoskeleton pathway (D)). DEG, differentially expressed genes; GSEA, gene set enrichment analysis; ROS, reactive oxygen species.

**Figure S2.** Analysis of human colon tissues. (A) *UCP2* mRNA levels in colon of Control group and IBD group. (Control n = 20, IBD n = 16). (B) L-LA levels in colon of Control group and IBD group. (Control n = 3, IBD n = 3). (C) Representative image of H&E staining of colon section from healthy controls and IBD patients. Scale bar is 500 µm and 100 µm. (n = 3). (D) Representative image of TEM observation of Control group and IBD group. Scale bar is 1 µm. (Control n = 3, IBD n = 3). UCP2, uncoupling protein 2; IBD, inflammatory bowel disease; L-LA, L-lactic acid; H&E, hematoxylin and eosin; TEM, transmission electron microscopy.

**Figure S3.** Synthesis and characterization of OPR and SGF-OPR. (A) Schematic representation of OPR synthesis. (B) Zeta potential intensity of OPR and SGF-OPR. (n = 3). (C) Representative image of TEM observation of OPR and SGF-OPR. Scale bar is 20 nm. (n = 3). (D) XPS spectrum of OPR and SGF-OPR. (E) XPS spectrum of C1s in OPR. (F) XPS spectrum of N1s in OPR. (G) XPS spectrum of O1s in OPR. (H) XPS spectrum of C1s in SGF-OPR. (I) XPS spectrum of N1s in SGF-OPR. (J) XPS spectrum of O1s in SGF-OPR. (K) ·OH scavenging ability of different doses of SGF-OPR. (L) Comparison of H_2_O_2_ scavenging capacity of OPR and SGF-OPR. (M) Comparison of O_2_·^−^ scavenging capacity of OPR and SGF-OPR. OPR, oral proton-reprogrammed nanomedicine; SGF, simulated gastric fluid; XPS, x-ray photoelectron spectroscopy.

**Figure S4.** Color change of OPR under SIF and SGF conditions. OPR was incubated in SIF or SGF for 0, 24, 48, 72, 96, 120, 144, and 168 hours to evaluate the extent of GP release according to the color change. SIF, simulated intestinal fluid.

**Figure S5.** Half-life calculation of OPR under SIF condition. The data of GP release (OPR degradation) from Figure 1J was used to generate a non-linear fitting curve and the corresponding half-life of OPR.

**Figure S6.** Targeting ability of OPR. (A-B) Synthetic schematic of OPR-FITC (A) and fluorescence spectrum of OPR and FITC-OPR (B). (C) Zeta potential of OPR-FITC and OPR. (n = 3). (D) Schematic representation of drug retention through histological examination after different administration routes (oral and intravenous injection) in IBD and healthy mice. (E) quantification of Figure 1K. (F) quantification of Figure 1L. (G-H) Comparison of fluorescence intensity accumulation of OPR in IBD lesions and healthy intestine and H&E staining results between IBD and healthy intestine tissue slices (red line: IBD lesion, blue line: healthy intestine) (G) and fluorescence intensity quantification (H). Scale bar is 2 mm, 500 µm, 300 µm respectively. (n = 3). (I) Representative image of immunofluorescent staining of MUC2 and TF between Control group and IBD group. Scale bar is 100 µm. (n = 3). (J) Representative fluorescent images of OPR-FITC adhesion on the surface of uncoated, mucin or transferrin coated polystyrene. Scale bar is 100 µm. (n = 3). (K) The binding energy of OPR/GP and MUC2/TF determined by molecular docking. FITC, fluorescein isothiocyanate; MUC2, mucin 2; TF, transferrin.

**Figure S7.** Distribution of GP-FITC in IBD and healthy mice after oral administration. Representative image of fluorescence *in vivo* of main organs (heart, liver, spleen, lung, kidney and colon) in IBD and healthy mice after oral administration of GP-FITC. Scale bar is 5 mm and 10 mm. (n = 3). GP, genipin.

**Figure S8.** Characterization of GPA. (A) Zeta potential of OPR and GPA. (n = 3). (B) Representative fluorescent images of GPA-FITC adhesion on the surface of uncoated, mucin or transferrin coated polystyrene. Scale bar is 100 µm. (n = 3). (C) Representative image of fluorescence *in vivo* of main organs (heart, liver, spleen, lung, kidney and colon) in IBD and healthy mice after oral administration of GPA-FITC. Scale bar is 5 mm and 10 mm. (n = 3). (D) Comparison of fluorescence intensity accumulation of GPA in IBD lesions and healthy intestine and H&E staining results between IBD and healthy intestine tissue slices (red line: IBD lesion, blue line: healthy intestine). Scale bar is 2 mm, 500 µm, 400 µm respectively. (n = 3).

**Figure S9.** Dosage screen of OPR based on the body weight change of mice after treatment. The body weights of healthy mice treated with 0, 2.5, 5, 10, 20, 40, and 80 mg/kg OPR were tracked for 7 days to screen the safe dosage range. (n = 5). Data represent means ± S.D. Statistical significance was calculated by one-way ANOVA with Tukey’s post-test, ^***^*p* < 0.01 *vs* Control.

**Figure S10.** Dosage screen of OPR based on the colon length change of mice after treatment. Gross specimen of colon length comparison among different groups. (n = 6).

**Figure 11.** Biocompatibility of OPR. (A) Effects of different concentrations of OPR on the viability of HT29 cells at 24 and 48 hours. (n = 3). (B) Schematic diagram of OPR toxicity test in mouse. (C) Body weight changes with time in mice given PBS and OPR orally. (n = 6). (D-G) Comparison of liver function (D-E) and kidney function (F-G) indicators in different groups. (n = 6). (H-J) Comparison of hematological parameters in different groups. (n = 6). (K) H&E staining of major organs (heart, liver, spleen, lung, kidney and colon) in different groups. Scale bar is 300 µm. (n = 6). Data represent means ± S.D. Statistical significance was calculated by one-way ANOVA with Tukey’s post-test.

**Figure S12.** The inhibition effect of OPR and GP on TcdB. Dose-response curves of OPR and GP were generated based on the cell viability assay data and the corresponding IC_50_ of OPR and GP on TcdB effect was calculated. (n = 3).

**Figure S13.** The effect of OPR on cell energy metabolism. (A) OCR assay in HT29 cells. (n = 3). (B) ECAR assay in HT29 cells. (n = 3). OCR, oxygen consumption rate; ECAR, extracellular acidification rate.

**Figure S14.** Analysis of metabolic indicators in mouse colon. (A-D) NAD^+^/NADH ratio (A), ATP/ADP ratio (B), Hexokinase 2 levels (C), and PFK activity (D) in mouse colon tissue. (n = 3). Data represent means ± S.D. Statistical significance was calculated by one-way ANOVA with Tukey’s post-test. ^###^*p* < 0.001 *vs* Control, ^*^*p* < 0.05 *vs* IBD, ^**^*p* < 0.01 *vs* IBD, ^***^*p* < 0.001 *vs* IBD. (ns: no statistical difference compared to IBD). NAD^+^/NADH, oxidized/reduced nicotinamide adenine dinucleotide; PFK, 6-phosphate fructokinase.

**Figure S15.** Concentration screen of OPR based on the regulation effect of cell viability. CCK8 assay was used to evaluate the effect of different concentrations of OPR in H_2_O_2_-induced HT29 cells (n = 3). Data represent means ± S.D. Statistical significance was calculated by one-way ANOVA with Tukey’s post-test, ^###^*p* < 0.001 *vs* Control, ^***^*p* < 0.001 *vs* H_2_O_2_. (ns: no statistical difference compared to H_2_O_2_). CCK8, cell counting kit 8.

**Figure S16.** Concentration screen of OPR based on the effect of ROS. Representative immunofluorescence images of DCFH-DA staining in HT29 cells under different treatment conditions. Scale bar is 250 µm. (n = 3). DCFH-DA, 2',7'-dichlorodihydrofluorescein diacetate.

**Figure S17.** Effect of OPR on mitochondria protection. Flow cytometry analysis of DCF (A), MitoSox (B), and JC-1 (C) in HT29 cells. (n = 3).

**Figure S18.** Colocalization analysis of OPR-FITC and mitochondria. Representative immunofluorescence staining images and co-localization analysis of OPR-FITC (green) and Mitotracker (red) in HT29 cells. Scale bar is 10 µm. (n = 3).

**Figure S19.** Interaction analysis between OPR and mitochondrial outer membrane proteins. (A-D) Interaction between OPR or GP and mitochondrial outer membrane proteins (TOM20, TOM34, VDAC1, VDAC2, TOM70, HSP90) (left) and imaging diagram (right) and bar chart of binding energy (D). (E) MDA levels in colon tissue from each group. (n = 3). Data represent means ± S.D. Statistical significance was calculated by one-way ANOVA with Tukey’s post-test, ^###^*p <* 0.001 *vs* Control, ^***^*p* < 0.001 *vs* IBD. (ns: no statistical difference compared to IBD). TOM20, translocase of outer mitochondrial membrane 20; TOM34, translocase of outer mitochondrial membrane 34; VDAC1, voltage-dependent anion channel 1; VDAC2, voltage-dependent anion channel 2; TOM70, translocase of outer mitochondrial membrane 70; HSP90, heat-shock protein 90; MDA, malondialdehyde.

**Figure S20.** Confirmation of UCP2-OE transfection in HT29 cells. (A) Representative images of Cy3 labelled UCP2-OE plasmid after transfection into HT29 cells for 8 hours. Scale bar is 50 µm. (n = 3). (B) *UCP2* gene expression in Control and UCP2-OE transfected HT29 cells after 24 hours of transfection. (n = 3). (C) UCP2 protein expression in Control and UCP2-OE transfected HT29 cells after 48 hours of transfection. (n = 3). Data represent means ± S.D. Statistical significance was calculated by unpaired two-tailed Student’s t-test. *^**^p* < 0.01, *^***^p* < 0.001. Cy3, cyanine 3; UCP2-OE, UCP2-over expression.

**Figure S21.** Effect of OPR in UCP2-OE transfected HT29 cells. (A) Representative image of immunofluorescence staining of DCFH-DA, JC-1, and MitoSox in HT29 cells under different treatments. Scale bar is 20 µm. (n = 3). (B) Representative image of flow cytometry of DCFH-DA, JC-1, and MitoSox in HT29 cells under different treatments. (n = 3).

**Figure S22.** Effect on ROS clearance of different drugs in mouse colon. Representative DHE staining image of colon tissues of each group. Scale bar is 200 µm. (n = 3). DHE, dihydroethidium.

**Figure S23.** Anti-apoptosis effect of OPR. (A-B) Representative images of TUNEL ﬂuorescence staining among diﬀerent groups and corresponding quantification. Scale bar is 200 µm. (n = 3). (C) Representative live/dead staining images of HT29 cells after different treatment. Scale bar is 20 µm. (n = 3). (D) Annexin V-FITC/PI flow cytometry assay of HT29 cells with different treatments. (n = 3). Data represent means ± S.D. Statistical significance was calculated by one-way ANOVA with Tukey’s post-test, ^###^*p* < 0.001 *vs* Control; ^***^*p* < 0.001 *vs* IBD. (ns: no statistical difference compared to IBD). TUNEL, terminal deoxynucleotidyl transferase dUTP nick end labelling; PI, propidium iodide.

**Figure S24.** Effect of OPR on pyroptosis pathway. (A) Quantification of HMGB1 in Figure 5G. (B) Quantification of C-Casp3 in Figure 5G. (C) Quantification of GSDME in Figure 5G. Data represent means ± S.D. Statistical significance was calculated by one-way ANOVA with Tukey’s post-test, ^***^*p* < 0.001.

**Figure S25.** Effect of different drugs on UCP2 expression in mouse colon. Quantification of Figure 5L. Data represent means ± S.D. Statistical significance was calculated by one-way ANOVA with Tukey’s post-test, ^###^*p* < 0.001 *vs* Control; ^*^*p* < 0.05, ^**^*p* < 0.01 *vs* IBD. (ns: no statistical difference compared to IBD).

**Figure S26.** Effect of different drugs on BAX and C-Cyt C expression in mouse colon. (A-B) Representative immunofluorescent staining images of BAX protein in colon tissue from different groups and corresponding quantification. (C-D) Representative immunofluorescent staining images of C-Cyt C protein in colon tissue from different groups and corresponding quantification. Scale bar is 200 µm. (n = 3). Data represent means ± S.D. Statistical significance was calculated by one-way ANOVA with Tukey’s post-test, ^###^*p* < 0.001 *vs* Control; ^***^*p* < 0.001 *vs* IBD. (ns: no statistical difference compared to IBD). BAX, BCL2 associated X; C-Cyt C, cytosolic cytochrome complex.

**Figure S27.** Effect of different drugs on junction proteins in mouse colon. (A-B) Representative immunofluorescent staining images of Occludin protein in colon tissue from different groups and corresponding quantification. (C-D) Representative immunofluorescent staining images of E-cadherin protein in colon tissue from different groups and corresponding quantification. (E-F) Representative immunofluorescent staining images of ZO-1 protein in colon tissue from different groups and corresponding quantification. Scale bar is 200 µm. (n = 3). Data represent means ± S.D. Statistical significance was calculated by one-way ANOVA with Tukey’s post-test, ^###^*p* < 0.001 *vs* Control; ^***^*p* < 0.001 *vs* IBD. (ns: no statistical difference compared to IBD).

**Figure S28.** Effect of different drugs on dsDNA release in mouse colon. Colocalization analysis of Figure 6J. (n = 3). dsDNA, double-stranded DNA.

**Figure S29.** Effect of different drugs on inflammatory cytokine levels in mouse colon. (A-C) The levels of TNF-α (A), IL-1β (B), and IL-6 (C) in the intestinal tissues of mice in different groups. (n = 3). Data represent means ± S.D. Statistical significance was calculated by one-way ANOVA with Tukey’s post-test, ^###^*p <* 0.001 *vs* Control, ^***^*p* < 0.001 *vs* IBD. (ns: no statistical difference compared to IBD). TNF-α, tumor necrosis factor-alpha; IL-1β, interleukin 1 beta; IL-6, interleukin 6.

**Figure S30.** Effect of different drugs on MPO activity in mouse colon. MPO activity levels in colon tissue homogenates from each group was evaluated with an MPO assay kit. (n = 3). Data represent means ± S.D. Statistical significance was calculated by one-way ANOVA with Tukey’s post-test, ^###^*p* < 0.001 *vs* Control, ^***^*p* < 0.001 *vs* IBD. (ns: no statistical difference compared to IBD). MPO, myeloperoxidase.

**Figure S31.** Analysis of human single-cell sequencing database. (A) The t-SNE plot of *UCP2* expression across different cell types in the colon of IBD patients and healthy controls. (Control n = 13, IBD n = 13). (B) The t-SNE plot of *GSDME* expression across different cell types in the colon of IBD patients and healthy controls. (Control n = 13, IBD n = 13). (C) The t-SNE plot of *STING* expression across different cell types in the colon of IBD patients and healthy controls. (Control n = 13, IBD n = 13).

**Figure S32.** Analysis of mouse colon transcriptomic sequencing data. (A) Correlation heatmap of gene expression among samples of mouse intestinal tissue transcriptome sequencing (Control n = 3, IBD n = 3, OPR n = 3). (B) Venn diagram showing the number of DEGs between Control, IBD, and OPR group of mouse intestinal tissue samples. (Control n = 3, IBD n = 3, OPR n = 3). (C) KEGG signal pathway enriched bubble diagram of DEGs between IBD group and OPR group (FDR < 0.05). (IBD n = 3, OPR n = 3).

**Figure S33.** Effect of OPR on the gene expression in mouse colon. mRNA levels of *UCP2* (A), *GSDME* (B), and *STING* (C) in mouse colon tissue were confirmed with qPCR. Data represent means ± S.D. Statistical significance was calculated by unpaired two-tailed Student’s t-test. ^*^*p* < 0.05, ^**^*p* < 0.01,^***^*p* < 0.001. (ns: no statistical difference). GSDME, gasdermin E; STING, stimulator of interferon genes.
